# Application of GUHA data mining method in cohort data to explore paths associated with premature death: a 29-year follow-up study

**DOI:** 10.1186/s12874-025-02477-6

**Published:** 2025-01-27

**Authors:** Lily Nosraty, Esko Turunen, Saila Kyrönlahti, Clas-Håkan Nygård, Prakash KC, Subas Neupane

**Affiliations:** 1 Faculty of Social Sciences, Centre of Excellence in Research on Ageing and Care, University of Helsinki, Helsinki, Finland; 2 Faculty of Social Sciences (Health Sciences) and Gerontology Research Center (GEREC), Tampere Universities, Tampere, Finland; 3 Faculty of Information Technology and Communication Sciences, Tampere Universities, Tampere, Finland; 4 Faculty of Social Sciences (Health Sciences), Tampere Universities, Tampere, Finland

**Keywords:** Midlife antecedents, General Unary Hypothesis Automaton (GUHA) method, Data mining, Mortality

## Abstract

**Background and Method:**

This study set out to identify the factors and combinations of factors associated with the individual’s premature death, using data from the Finnish Longitudinal Study on Ageing Municipal Employees (FLAME) which involved 6,257 participants over a 29-year follow-up period. Exact dates of death were obtained from the Finnish population register. Premature death was defined as a death occurring earlier than the age- and sex-specific actuarial life expectancy indicated by life tables for 1981, as the baseline, with the threshold period of nine months. Explanatory variables encompassed sociodemographic characteristics, health and functioning, health behaviors, subjective experiences, working conditions, and work abilities. Data were mined using the General Unary Hypothesis Automaton (GUHA) method, implemented with LISp-Miner software. GUHA involves an active dialogue between the user and the LISp-Miner software, with parameters tailored to the data and user interests. The parameters used are not absolute but depend on the data to be mined and the user’s interests.

**Results:**

Over the follow-up period, 2,196 deaths were recorded, of which 70.4% were premature. Seven single factors and 67 sets of criteria (paths) were statistically significantly associated with premature mortality, passing the one-sided Fisher test. Single predicates of premature death included smoking, consuming alcohol a few times a month or once a week, poor self-rated fitness, incompetence to work and poor assured workability in two years’ time, and diseases causing work disability. Notably, most of the factors selected as single predicates of premature mortality did not appear in the multi-predicate paths. Factors appearing in the paths were smoking more than 20 cigarettes a day, symptoms that impaired functioning, past smoking, absence of musculoskeletal diseases, poor self-rated health, having pain, male sex, being married, use of medication, more physical strain compared to others, and high life satisfaction, intention to retire due to reduced work ability caused by diseases and demanding work. Sex-specific analysis revealed similar findings.

**Conclusion:**

The findings indicate that associations between single predictors and premature mortality should be interpreted with caution, even when adjusted for a limited number of other factors. This highlights the complexity of premature mortality and the need for comprehensive models considering multiple interacting factors.

**Supplementary Information:**

The online version contains supplementary material available at 10.1186/s12874-025-02477-6.

## Background

Average life expectancy has continued to increase in recent years, but many premature deaths still occur. The National Cancer Institute defines premature death as death that occurs before the average age of death in a certain population, or more specifically before an individual’s age- and sex-specific life expectancy [1]. Mortality is a key indicator of population health, and factors associated with premature mortality have received major attention in the scientific literature.

Earlier studies have identified several factors linked to premature mortality, including adverse socioeconomic status, physical and mental diseases and disabilities, various risk behaviors, and adverse work-related factors across the life course [2–10]. Smoking and physical inactivity in midlife are well-established risk factors for premature mortality [2, 4, 11]. Poor work ability in midlife and old age [5, 6], high physical strain at work [6], sedentary work [6], a hazardous work environment [7, 8], and job-related psychosocial factors such as poor social support and lack of perceived control [9] have also been linked to premature mortality. The Job-Demand-Control-Support model, in particular, has highlighted the association of stress with job demands [10]. The model shows that a low level of job control increases the risk of death [11] and that low work support is a significant predictor of premature mortality among women [12]. Low job control and low support also increase the mortality risk [13]. The role of various psychological factors and social relations during the life course is also well-established [14]. Psychological stress at work is known to contribute to mortality [15].

Most previous studies have sought to identify risk factors for premature mortality within and between different domains (e.g., social, physical, and psychological) and in isolation from each other, based on prior hypotheses [16]. However, there are likely several important predictors of premature mortality with separate or joint contributions. If the analysis focuses on a single predictor and disregards other associated factors, its effect size is bound to be exaggerated, even if the model is adjusted for limited factors [17]. It is therefore important to incorporate predictors from across different life domains and to use a method that allows us to find the hypothetical combination of factors contributing to premature mortality.

In this study, we used the population life Table [21] to calculate premature death for each deceased participant. We hypothesize that premature death results from the interplay of multiple midlife antecedents. We examined a wide range of midlife factors from a sample of municipal employees to predict individual-based premature mortality based on age and sex during a 29-year follow-up period. Our research question was: which factors and their combinations are associated with the individual’s premature death? We addressed this question by using the *General Unary Hypothesis Automaton* (GUHA) data mining method, which imposes no particular structure or assumptions on the prediction model.

## Method

### Data sources

We used data from a prospective follow-up of the Finnish Longitudinal Study on Ageing Municipal Employees (FLAME) [18] conducted among a representative sample of the largest municipal occupational groups in Finland. The data were collected using structured questionnaires that were sent to 7,344 municipal employees aged 44–58 years at baseline in 1981. Responses were received from 6,257 employees (44.7% men) who had worked in the municipal sector for at least five years, giving a response rate of 85.2% [18].

Exact dates of death were obtained from the Finnish national population register. The mortality information was linked to the questionnaire data using personal identification codes. Vital status of the study participants was ascertained up to March 31, 2010.

### Measures

#### Premature mortality measure (outcome variable)

An individual measure of premature mortality was calculated based on each participant’s age and sex and the population life table. Two elements were used in measuring premature death: the number of years each person survived after baseline (1981), which was obtained from the population register, and actuarial life expectancy (ALE) at baseline, which was based on each person’s sex and age. Finland’s ALE for the baseline year 1981 was obtained from human mortality data [19]. We then calculated premature death by subtracting the number of years lived after baseline from actuarial life expectancy in 1981. If a participant died before their actuarial life expectancy, that was defined as a premature death. A death occurring less than nine months before actuarial life expectancy was not considered premature. This threshold was chosen to avoid overclassification due to minor discrepancies or measurement limitations that do not necessarily indicate premature mortality. To assess the robustness of the analysis, sensitivity analyses were conducted using two alternative buffer periods of 3 and 6 months.

#### Predictors of premature mortality (explanatory variables)

##### Individuals’ characteristics

The individual characteristics examined comprised a total of 80 variables. Sets of variables were drawn from the domains of sociodemographic characteristics, health and functioning, health behavior, subjective experiences and feelings, work conditions and work ability. The variables are described in detail in the Appendix, Table A. Table A presents a comprehensive overview for the description of the factors used, how they were recoded to use in the analysis, and the percentage of the categories for each factor.

*Sociodemographic characteristics* included age, sex, marital status, years of full-time education, social class (based on main occupation), type of work contract, satisfaction with pay level, self-rated household standard of living, financial situation, pension security, and age at entry into paid employment.

*Health and functioning variables* included self-rated health, self-rated physical fitness (as compared to others of the same age), assured ability to work in two years, body mass index (BMI) (kg/m2), self-reported diagnosed diseases, medication use, symptoms that impair functioning, diseases that affect daily life, diseases causing work disability, musculoskeletal diseases, any pain (in neck, shoulders, elbow, wrist, fingers, lower back, thighs, ankle or foot), pain that interferes with work, psychosomatic symptoms, quality of sleep, sleeping difficulties, and memory problems.

*Health behavior variables* included past smoking, current smoking (as indicated by the tobacco index), leisure-time physical activity, and alcohol consumption.

*Subjective experiences variables* included feelings such as life satisfaction, satisfaction with social relationships, satisfaction with financial situation, ability to enjoy daily activities, feeling nervous, feeling dizzy, feeling depressed, feeling hopeful, feeling active and energetic, anxiousness, feeling reluctant, interrupted thoughts, wishing to work when absent from work, wanting to skip work, enjoying work, and loss of a close friend.

*Work-related variables* included working in a warm or cold environment, dry or humid environment, dirty environment, polluted environment, noisy environment, working with harmful substances, restless working environment, mentally demanding work, physically demanding work, repetitive movements, standing still, being seated in the same place for extended periods, awkward working postures, walking or moving a lot at work, carrying and lifting heavy objects, interaction at work, responsibility for others at work, problems at work due to high work responsibility, time pressure at work, excessive control at work, forced pace of work, and isolation or loneliness at work. We also examined the individual’s influence over the work environment, willingness to change jobs, changing jobs due to diseases, number of work absences due to health reasons, work ability compared to two years ago, work ability index, physical work ability, mental work ability, current workload compared to previous year, overtime work, irregular working hours, working hours format, difficulties in relationship due to working hours, difficulties in leisure activities due to working hours, tiredness due to working hours, nervousness due to working hours, duration of commute to work, and self-rated physical fitness. Also, the participants’ intention to retire due to different reason, mental strain at work, physical strain at work, reduced work ability due to diseases, employment situation, and changes in work assignments were included in the analysis.

### Main features of GUHA data mining method

Data mining is a process aimed at finding interesting relations, associations and structures in a given dataset. There are several different types of data mining methods, designed and developed for different types of problems. Many of them are based on statistics, neural networks, or machine learning techniques based on various artificial intelligence applications [20]. The General Unary Hypothesis Automaton (GUHA) method [21–23] differs from mainstream data mining methods in that it is based on logic formalism. Statements on associations are labeled as TRUE or FALSE. Those labeled as TRUE are called *hypotheses*,* GUHA-assoc*, or *path* which refer to the associations that are supported by data [24]. In this study, we have chosen to use the word “*path*”. In the GUHA context, “data” is a flat matrix with rows and columns. Thus, when we talk about ‘data’, we mean this data matrix. In principle, its cells can contain any form of symbols, but in practice, the data must be converted to binary form before the data mining process can take place. In practice, using GUHA in mining data is an active dialogue between the user and the LISp-Miner software, the computer implementation of the GUHA. The parameters used are not absolute but depend on the data to be mined and the user’s interests. A particular strength of the GUHA method is that it allows us to systematically investigate small but significant dependencies in a large data mass [22]. The GUHA method is a powerful approach used in exploratory data analysis and the discovery of associations within data [21–23]. The GUHA method is a structured approach to hypothesis generation and testing, leveraging associative logic to automate the discovery of significant patterns within data. Its ability to handle large hypothesis spaces and provide formalized testing makes it a valuable tool in exploratory data analysis by highlighting the associations and not establishing causal relationships [21–23]. This capability makes GUHA particularly valuable in epidemiological research. Unlike many machine learning models, GUHA provides transparent and interpretable results [21–23], making it an ideal tool for exploratory analysis and hypothesis generation in our study.

LISp-Miner software package has been used for analysis.

Our *analytical question* was as follows: “Which factors (or predicates in GUHA terminology) and factor-category combinations (multi predicates in GUHA terminology) are associated with the individual’s premature death?” LISp-Miner detects all dependencies relevant to the question. The analytical ability of LISp-Miner software is based on GUHA’s specific logical language, the central part of which are generalized quantifiers involving statements φ and ψ. Here, φ and ψ represent logical statements describing specific conditions within the data. For example, one quantifier might describe how often “φ is followed by ψ” or that “φ and ψ almost always exclude each other,” with both φ and ψ being defined by the user in context.

The generalized quantifiers are not absolute measures and depend on the current data and are defined by the user. For example, the *quantifier more often than average* can sometimes mean at least ten times more often or just twice as often as on average. Here, φ and ψ are logic statements describing the data. For example, φ could mean “Individual X is a married male with a BMI of 25 to 30” and ψ “Individual X died prematurely”. In GUHA language, “Individual X is married”, “Individual X is male”, etc. are called *predicates*. Each variable (*attribute)* is divided (by the user’s choice) into several predicates; for example, the attribute ‘BMI’ is divided into four predicates BMI (20 < ), BMI (20 - <25), BMI (25 - <29), BMI (30 -).

There are usually hundreds of such predicates. In this study the 80 attributes analyzed were divided into two to four predicates each, yielding a total of 333 predicates and thousands of logical combinations whose association with premature death was investigated. It is the task of LISp-Miner to find statements with the value TRUE. By statements, we mean logical combinations of predicates (denoted by φ) associated with the predicate premature death(yes), (denoted by ψ).

Within the user-specified boundary conditions, LISp-Miner checks contingency tables of the form.

Giving that m is the number of rows in the (Boolean) data matrix (6265 in this study) and.

- a is the number of objects satisfying both φ and ψ,
- b is the number of objects satisfying φ but not ψ,
- c is the number of objects not satisfying φ but satisfying ψ, and.
- d is the number of objects not satisfying φ nor ψ (see Table 1).

**Table 1:** A contingency table produced by a boolean-valued data matrix

	ψ	not-ψ	
φ	a	b	**r** = a + b
not-φ	c	d	**s** = c + d
	**k** = a + c	**l** = b + d	**m** = a + b + c + d

We used the *Above Average Quantifier* and *Fisher Quantifier* because the paths they produce answer this question: In terms of combinations of predicates in φ, which are most strongly related to the predicate ψ (i.e., premature death). In addition, such relations (statements denoted by φ ≈ ψ) are statistically significant according to Fisher’s test that is, in the LISp-Miner procedure, they pass the classic Fisher’s one-sided exact test.

Given a truth valuation function *v* which maps statements to the set {TRUE, FALSE}, then in the case of the Above Average Quantifier, the truth definition *v* (φ ≈ ψ) = TRUE is given by the formula

$$\:\frac{a}{a+b}\ge\:\frac{(1+p)(a+c)}{m},\:\text{w}\text{h}\text{e}\text{r}\text{e}\:p>\:0\:\text{a}\text{n}\text{d}\:Base\:\le\:a$$

and *v* (φ ≈ ψ) = FALSE elsewhere in the related contingency table. For example, if *p* = 4 and the above two conditions hold, then the statement “ψ is at least 5 (= *p* + 1) times more common for individuals satisfying φ than it is on average” is supported by the data. In other words, “Among objects satisfying φ there are at least 400% more objects satisfying ψ than there are objects satisfying ψ in the whole data matrix”.

In connection with the Above Average Quantifier, the parameter *(p)* or frequency coefficient expresses the prevalence of that association, the magnitude of the dependence compared to the mean. For example, if frequency coefficient (*p*) is 2, then premature death is (*p* + 1) 3 times more common among those with the characteristic under consideration than in the entire study population. Generally, the higher the value of the parameter *p*, the more plausible the statement.

On the other hand, the *Base* value (also called *support*) should also be large enough, otherwise the result will have low general significance. *Base* determines the minimum number of cases where predicate and outcome should occur in the data at the same time. In the contingency table, this value is marked with the symbol *a*. A necessary condition for a certain dependency to be valid in the data is that a is at least the size of *Base*. In this study the *Base* values ​​ranged from 75 to 400 and the *p* values ​​from 2.2 to 0.2 for all participants. The *Base* value for women was considered 50 and 30 for men.

For the Fisher Quantifier, the truth definition *v* (φ ≈ ψ) = TRUE is given by the condition $\:ad>bc$ and

$$\sum\nolimits_{i = 1}^{min\{ b,c\} } {\frac{{r!s!k!l!}}{{m!\left( {a + 1} \right)!\left( {b - 1} \right)!\left( {c - 1} \right)!\left( {d + 1} \right)!}}\, \leqslant \,\alpha \,\,{\text{where}}\,0 < \alpha \, \leqslant \,0.5}$$

The lower the value of 𝛼, the less likely it is that the association is due to chance. In this study, we used the value 𝛼=0.001.

LISp-Miner verifies up to hundreds of thousands of contingency tables generated by the data but prints only those labelled as TRUE. However, due to the strong logical and combinatorial basis, LISp-Miner does not examine all possible contingency tables but only those relevant to the question.

GUHA employs an automated process to explore combinations of predicates and logical relationships within the dataset. It systematically generates and tests hypotheses that involve multiple predicates to identify statistically significant patterns or associations [21–23]. GUHA method systematically discovers multi-predicates by formulating hypotheses in a logical framework that combines multiple variables or predicates using logical operators. GUHA automates the exploration of these hypotheses, evaluating them based on statistical measures like support to identify significant patterns within the data. The method’s flexibility in varying the *Base* parameter allows it to adapt to different dataset characteristics and hypothesis spaces, facilitating the exploration of diverse sets of potential associations. This approach not only enhances the discovery of complex associations that may be missed by traditional methods but also, optimizes computational efficiency by focusing on promising areas of the hypothesis space [21–23].

### Performing GUHA analysis

In order to find single and multiple predicates’ significant associations and dependencies of factors (attributes) with premature death, analyses were conducted among all participants and separate analyses were performed for women and men. The current LISp-Miner version allows us to search for combinations of a maximum of five predicates as multi-predicates’ associations. The distribution of premature mortality was not normal.

To find single predicates with significant associations, we first set the *Base* value at 50 and used the value *p* = 0.5 and Fisher Quantifier 𝛼=0.001 for all participants and men. For women, the *Base* value was reduced to 30 with the same *p* and Fisher Quantifier (since we were unable to find any predicate with a *Base* value of 50).

To find multi-predicates, paths to premature mortality, we set the *Base* value at 700, parameter *p* = 0.55 and Fisher Quantifier 𝛼=0.001 because there were 1545 premature deaths in the data. However, after testing about 5 million contingency tables (verifications), no path was found. When the *Base* value was reduced to 400 with the same parameter *p*, seven paths out of 18 million possible verifications were found (Task 1). When the *Base* value was reduced to 300 and the value *p* = 0.7 with over 34 million verifications, 11 paths were recognized (Task 2). The *Base* value was reduced to 200 with *p* = 1 for the Above Average Quantifier with over 64 million verifications, and seven paths were found (Task 3). To find better-justified paths, we further reduced the *Base* value to 150, 100, and 75 (Tasks 4, 5 and 6). The highest values of parameter *p* for the Above Average Quantifier were *p* = 1.2, 1.4, and 1.5, respectively. This yielded about 9, 151 and 227 million verifications with 6, 21, and 15 paths, respectively (Tasks 4, 5 and 6). In the entire process of searching for multi-predicates, we found 67 paths containing unique predicates not emerging in analyses of single predicate associations. We further applied Bayesian statistical methods to interpret the patterns and regularities in more detail [25], utilising posterior probability distributions of generalised quantifier parameters.

Bayesian analysis of the identified paths is also available while conducting the GUHA analysis. Bayesian analysis of the paths indicated a high level of certainty, around 99%, and that premature mortality is 1.43 to 2.16 times more prevalent among individuals who fulfil the criteria outlined in the Appendix for each path.

The same analyses were performed among women and men separately. For these separate analyses we had to reduce (because of stratification) the *Base* value to 100, with the Fisher Quantifier at 𝛼=0.001. First, we searched for individual predicates related to premature death. We then searched for five predicates that together were possibly associated with premature death. In this analysis we used *Base* = 100, Fisher Quantifier 𝛼 = 0.001 and Above Average Quantifier *p* = 1 for both sexes (with 50 million contingency tables for men). Since we were unable to find any paths for women, we reduced *p* to 0.6 with 74 million contingency tables. The three paths with combinations of five predicates for men and 12 paths for women, were found to be statistically significant and related to premature death. The ‘a’ value (Table A) is 100–112 for women and 103–170 for men. The *Base* value was at least 50, *p* = 0.9, and 𝛼=0.001 for men. For women, the analysis was conducted with the *Base* value = 100, *p* = 0.76 and 𝛼=0.001. All the findings were considered statistically significant, and each finding was 1 + *p* times more common than the average in the whole data.

## Results

The mean age at baseline was 50.5 years (Standard Deviation, SD 3.6) and 56.9% of participants were female. The mean age for women was 50.4 (SD 3.6) and men was 50.5 (SD 3.6). Of the total of 2,196 deaths recorded during the follow-up, 70.4% (*n* = 1545) were premature (with the threshold of 9 months). The differences in premature mortality frequency between buffer periods were 0.7% between 3 and 6 months and 0.6% between 6 and 9 months, as observed in sensitivity analyses. The total number of deaths among the 2797 male participants was 1333, with the share of premature deaths at 66.5% (*n* = 886). The corresponding figures for the 3460 women were 863 deaths and 88.4% (*n* = 763) premature deaths. The median life expectancy at baseline for our study population was 26.30 years, while the median years of follow-up for the deceased population was 20 years. The percentage of the categories for each factor used in the analysis is presented in the Appendix, Table A.

In the first step of analysis, we found eight predicates in the total study population that were significantly associated with premature death. All of them were supported by the Fisher Quantifier and the Above Average Quantifier (Table 2). Two predicates of smoking (smoking less than 20 cigarettes a day and more than 20 cigarettes a day), consuming alcohol a few times a month or once a week, poor self-rated fitness compared to others of the same age, assured inability to work in two years’ time, diseases causing work disability, and feeling dizzy often (only for men) showed frequency coefficients of over 0.50 (i.e., the *p* value of the Above Average Quantifier). Smoking and alcohol consumption were two predicates that had the highest *Base* value. An example of the interpretation of findings for the single predicates (Table 2) is;

**Table 2:** Single predicates of premature death in total population and both sexes

Variable	“a” value			Frequency coefficient ¹			Variable	“a” value			Frequency coefficient ¹		
	Total	Male	Female	Total	Male	Female		Total	Male	Female	Total	Male	Female
Smoking less than 20 cigarettes a day	236	-	-	0.54		-	Incompetence to work in two years’ time	97	56	41	0.65	0.57	0.68
Smoking more than 20 cigarettes a day	287	-	-	1.00		-	Diseases affecting work which cause a sense of being unable to work	85	50	35	0.98	0.61	1.19
Alcohol use a couple of times a month or once a week	249	-	-	0.51	-	-	Feeling dizzy often	-	82	-	-	0.61	-
Worse fitness strength compared to others at the same age	115	70	45	0.71	0.64	0.69							

¹ Frequency coefficient (*p*) expresses the prevalence of that association, the magnitude of the dependence compared to the mean. For example, if ‘frequency coefficient **(*p*)**’ is 2, then premature death is (*p* + 1) 3 times more common among those with the characteristic under consideration than in the entire study population

Among the 287 individuals who smoked more than 20 cigarettes a day, premature death is two (1 + *p*) times more common than the average for the population.

The second step of the analysis to find multi-predicates revealed 67 paths in the total study sample, 18 paths among women (with *Base* = 100) and three paths among men (with *Base* = 50) that were significantly associated with premature death. The first path for premature mortality (with *Base* = 700) included five predicates: no musculoskeletal diseases, previous smoking, symptoms that impaired functioning, regular shiftwork in the daytime or evening, and reduced work ability due to diseases. Premature mortality was 1.55 times more frequent among individuals who met those criteria than on average. The second and third paths overlapped greatly with the first path, and when pain was added to the second path, reduced work ability due to diseases was excluded from the path. In the third path, regular shiftwork in the morning or evening was replaced by male sex.

**Fig. 1:**
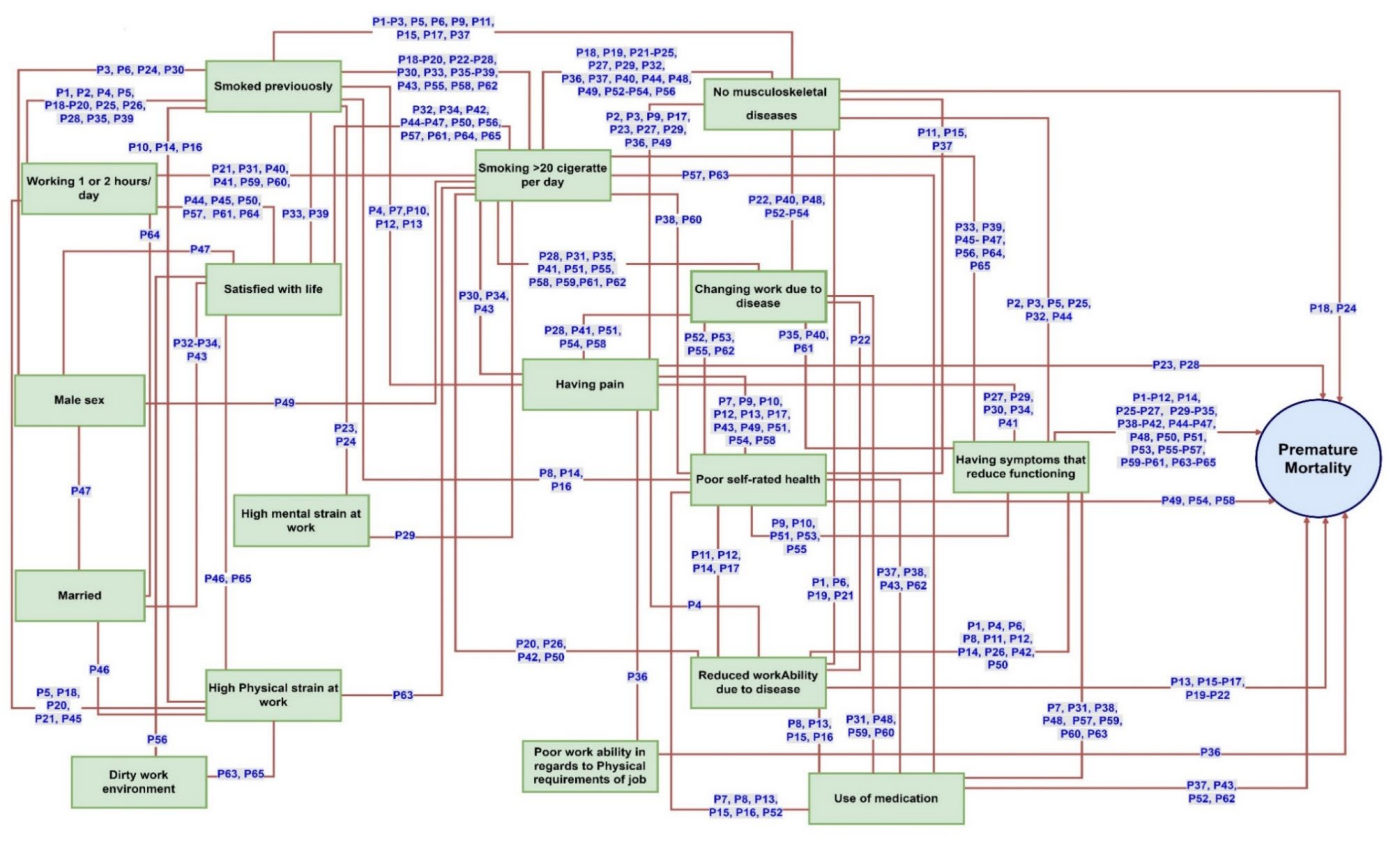
Paths to premature death. Each path is a combination of five predicates (identified by path (P) number) significantly associated with premature death (shown by connected lines). Description of the paths is available in appendix B

Only 17 predicates were present in the 67 paths to premature mortality found in the total study sample. Some of those predicates appeared repeatedly in many paths (Table 3). Smoking more than 20 cigarettes a day appeared in 49 paths, while symptoms that impaired functioning, previous smoking, no musculoskeletal diseases, one or two hours of work a day, poor self-rated health, and having pain appeared 46, 40, 29, 26, 23, and 22 times, respectively. Other predicates appearing in the paths were male sex, use of medication, life satisfaction, absence of diseases affecting work, being married, and having less physical fitness compared to others of the same age. Also, intention to retire due to reduced work ability as a result of diseases, and physically or mentally demanding work (Table 3) appeared in the paths. Figure 1 presents all paths to premature death.

**Table 3:** Predicates recognized in 67 paths of each task with different *base* and *p* values for the whole population

Predicates	Total study sample (Tasks with different Base and *P* value)^1^							Female 12paths	Male 3 paths
	Task 1 7 paths	Task 2 11 paths	Task 3 7 paths	Task 4 6 paths	Task 5 21 paths	Task 6 15 paths	Total 67 paths		
No musculoskeletal diseases	5	4	6	3	7	4	29	4	2
Smoked previously	7	11	6	5	8	3	40	11	2
Having symptoms that impair functioning	7	7		5	16	11	46	12	2
Working hours is 1–2 h	5	0	4	3	9	5	26		2
Intention to retire: reduced work ability due to diseases	4	8	4	1	2	0	19	6	
Having pain	3	6	1	4	5	3	22	7	1
Male sex	2	0	1	1	2	0	6		
Intention to retire: physical work strain	1	3	3		2	2	11	4	
Poor self-rated health		11			4	8	23		1
Use of medication		5			5	6	16	2	
Smoking more than 20 cigarettes			7	6	21	15	49		3
Intention to retire: mental strain at work			2	1	1	0	4	2	
Not changing work due diseases			1	1	5	8	16		2
Married					6	1	7		
Satisfied with life					10	5	15		
Worse fitness compared to others at the same age					1	0	1	1	
Detrimental factors at work: not dirty						3	3		
Not having conflict in their closed relationship								2	

^1^ Task 1: *Base* 400, and parameter *p* = 0.55, Task 2: *Base* 300, and parameter *p* = 0.70, Task 3: *Base* 200, and parameter *p* = 1, Task 4: *Base* 150, and parameter *p* = 1.2, Task 5: *Base* 100, and parameter *p* = 1.4, Task 6: *Base* 75, and parameter *p* = 1.5

The second-step analysis for women revealed several combinations of five predicates that were significantly associated with premature death. All of those combinations them were supported by the Fisher Quantifier and were above average. Many of the variables that appeared in the paths for women were present in paths for the total study population (Table 3). Having symptoms that impair functioning were observed in all twelve paths while smoked previously appeared in eleven paths. Having pain was recurred in more than half of the paths (seven paths). The intention to retire due to reduced work ability as a result of the diseases in six paths and physically demanding work in four paths accordingly. Use of medication, intention to retire due to mentally demanding work, and not having conflict in their closed relationship were appeared in two paths. The perception of worse fitness compared to others at the same age appeared in a single path only.

Three multi-predicate paths to premature death were found among men, and these paths overlapped. Smoking more than 20 cigarettes a day, past smoking, symptoms that impaired functioning, no musculoskeletal diseases, one or two hours of work a day, poor self-rated health, and having pain appeared in the paths. The highest *p* was 1.12 for the path which comprised individuals who had smoked previously and who were currently smoking more than 20 cigarettes a day with no musculoskeletal diseases but who experienced pain and had poor self-rated health.

### Sensitivity analysis

Sensitivity analyses using alternative buffer periods of 3 and 6 months were conducted using the quantifiers of *Base* 400 and *p* = 0.5, in addition to the *Base* 100 and *p* = 0.4 and above-average dependency. The number of identified paths increased with the 3-month threshold. While the paths identified in the models remained consistent, minor variations in decimal values, such as shifts in Average Absolute Difference (AAD) from 0.501 to 0.503, were observed. These analyses confirmed that the identified factors with minimal differences and significance remained robust across buffer periods.

## Discussion

This study utilized life table information to measure individual premature mortality in a 29-year follow-up among Finnish municipal employees in 1981. We applied the GUHA data mining method in two steps to identify single predicates and multi-predicate (paths) associated with premature mortality. In contrast to previous studies that have focused on analyzing specific associations [5, 7], we used the GUHA method to identify associations between certain predicates and premature death. It is important to note that our study population consists of a working cohort, which might have a better health profile than the general population. However, despite this, the premature death rate in this group was higher than expected indicated by the life table. Any data-supported significant associations between independent predicates and premature death were examined through GUHA analysis. The findings were significant in explaining variations in premature mortality rates during this period.

A major contribution of this study is identifying combinations of factors from different domains of life that are associated with premature mortality. Most of the factors (attributes) selected as single predicates are known predictors of premature mortality, yet with the exception of smoking they did not appear in the multi-predicate paths. This finding suggests that any associations discovered between one predictor with premature mortality must be interpreted with extra caution even if adjusted for the limited number of other factors [17]. The association of a single factor may be affected by the presence of other factors from the same or different domains of life. We found seven, four, and three single predicates in the total study sample, men, and women, respectively, that were associated with premature mortality. In addition, we observed 17 predicates that created 67 paths of predicate combinations that predicted premature mortality in the total study sample. It is remarkable that of the 80 possible attributes (which are further divided into 333 predicates), 65 attributes (316 predicates) did not appear to be significantly associated with premature death, at least in the clusters of five predicates. However, this does not rule out the possibility of some weaker link between these predicates and premature death.

Predicates that contributed to the paths were primarily from the behavioral, health, and sociodemographic domains. The self-perceived responses such as intention to retire and feelings related to the work environment also appeared in the paths, which may indirectly reflect the importance of well-being at work. The absence of musculoskeletal diseases, but having pain, symptoms that impaired functioning, poor self-rated health, and medication use appeared more often in the paths. Past smoking and currently smoking more than 20 cigarettes a day were among the most common predicates in the paths. Being married and male sex were two sociodemographic predicates that appeared in the paths but less frequently. Short daily working hours (1–2 h a day) was among the predicates that occurred repeatedly in many paths from work-related domains. The combination of short daily working hours with having symptoms that impaired functioning and past smoking increased the chance of premature death. The combination of predicates for the total study sample differed from the results of the sex-stratified analysis.

Our analysis confirmed the earlier finding that smoking more than 20 cigarettes a day (more than a pack) is a leading contributor to premature mortality. Smoking is a well-known predictor of the risk of mortality from all causes [26, 27]. In our study, as a single predicate, it increased the chance of premature death 2 times. Smoking less than 20 cigarettes a day also increased the chance of premature death 1.5 times. Smoking more than 20 cigarettes a day also appeared in most paths leading to premature mortality in the total study sample. The chance was also affected by the combination of smoking more than 20 cigarettes a day with other factors, as Fanelli & Ioannidis indicate inaccurate estimation of effect size with a single predictor [17]. However, the chance increased in most paths, contrary to the suggestion of Fanelli & Ioannidis that analysis of the single predictor can result in an exaggerated effect size for that predictor [17]. Past smoking and current smoking of more than 20 cigarettes a day combined with having pain or symptoms that impaired functioning were more common predicates in the paths to premature mortality, with high support in the total population. Past smoking and symptoms that impaired functioning appeared in all paths to premature death among women and were accompanied by having pain in more than half of the 11 paths found. Smoking more than 20 cigarettes a day did not appear in multi-predicate paths to premature death among women.

Satisfaction with life and being married appeared in some paths when we lowered the *Base* value in the total study sample and among men. Satisfaction with life also appeared more often in combination with having symptoms that impaired functioning and less working hours and past smoking or smoking more than 20 cigarettes a day. It is well-documented that dissatisfaction with life is a predictor of mortality [27, 28]. However, our analysis suggested that high life satisfaction appeared in some paths to premature mortality.

Life expectancy differs across time, and between age cohorts, as well as between men and women. Therefore, we used age- and sex-specific period life tables of the Finnish population from 1981 to define premature death and minimize the effect on our analysis. This eliminated the effect of sex at baseline, but the appearance of male sex in the paths to premature death in this analysis may suggest that even in midlife, it is possible that sex introduces a pathway for certain behaviors, work patterns and societal differences.

Being married was detected in seven paths. There are contradictory reports on the marital status and mortality. However, some earlier studies have shown that unmarried marital status is associated with adverse health and mortality outcomes [29, 30], and this association has been reported more frequently for men [30].

The variable describing the intention to retire due to “reduced work ability caused by diseases” appeared in 19 paths to premature death. This association has not been assessed in earlier studies, and it may reflect the attitudes and beliefs of individuals toward work and retirement. More than three-quarters of those harboring such intentions had more than three diseases. This finding raised the question of why these diseases were not identified as predictors of premature mortality in our analysis. It is worth noting, however, that diseases are not necessarily independent predictors since their effect may be mediated by other variables such as medication use, mobility limitations, assured inability to work in two years or the thought and expectations of declining health. These predictors can be understood as distal predictors of mortality that impact the likelihood of premature mortality through their associations with health, disease, and functioning. When measures defining these factors were included in the prediction models, the independent association of these distal predictors disappeared.

One of the predictors that emerged in our analysis, but has not been examined in earlier research is mental strain at work as a reason for early retirement. Another predicate in our study was an uncertain health outlook from the point of view of continued employment, reflecting the participants’ own assessment of their health status. This assessment may mediate information from the human body to individual consciousness and incorporate that information into these responses. This interpretation draws on the explanation offered by Jylhä on how self-rated health can predict mortality [31].

Another predictor appearing in the paths was poor self-rated health, one of the known predictors of mortality [31, 32]. It was accompanied by past smoking in all paths and either symptoms impairing mobility or medication use in the paths.

Absence of musculoskeletal diseases appeared in most paths to premature death, and was combined with having pain. The systematic review by Jackson et al. (2015) showed that the prevalence of chronic musculoskeletal pain in the general adult population and in older adults aged 65 years or over is 26% and 39%, respectively [33]. It is still debated whether chronic musculoskeletal pain is associated with a higher risk of mortality, possibly due to the definition of musculoskeletal pain applied in previous research [34, 35]. Li et al. [36] reported that having no musculoskeletal disorders could help offset some of the health risks associated with shift work. However, the potential problems of shift work, such as circadian rhythm disruption and stress, still pose significant risks for premature mortality. In our study, we were able to distinguish predicates for having musculoskeletal disease and having pain. Nonetheless, these findings could be attributed to multiple comparisons or a Type I error. We attempted to minimize this risk by using an extremely low significance threshold for the Fisher Exact test (*p* < 0.001), however, it was not diminished.

In addition to the findings that aligned with previous studies, we identified many novel associations with subjective factors related to feelings and satisfaction. This is the key benefit of the data mining approach, which allows us to identify previously unknown factors.

All the predicates selected by GUHA were significantly associated with premature death, but those significant associations should not be interpreted as causal relationship. One of the advantages of using the GUHA data mining method is that it paves the way for future investigations and uncovers more detailed information about the individuals meeting the common criteria in their group.

The GUHA data mining method is a data-driven approach particularly suited for exploratory data analysis, enabling automated generation and testing of multiple hypotheses without predefined assumptions. This method can uncover complex, non-linear, and non-parametric associations, providing a flexible and thorough way to explore patterns and relationships within the data [37]. However, GUHA has some limitations, such as the complexity and computational demands. The complexity can lead to longer processing time. Additionally, in observational studies, associations may be influenced by complex, unobserved factors or inverse causality [38], such as no musculoskeletal diseases or regular shift work, despite using a highly conservative significance threshold (𝛼 = 0.001) for Fisher’s exact test, which rigorously controlls for type I errors in contingency table analyses.

## Conclusion

The interplay of multiple midlife antecedents from various domains of life including sociodemographic characteristics, health and functioning, health behavior, working conditions, and subjective experiences determines premature death. Some of the key findings include the significance of smoking (particularly smoking more than 20 cigarettes a day), self-rated health, medication use, and the interplay between factors like musculoskeletal diseases, pain, and mobility limitations. Additionally, the study highlights the value of data mining methods like GUHA for uncovering complex and previously unknown associations, emphasizing the intricate web of influences on premature mortality. This study suggests a promising new direction for understanding how different midlife domains interact to shape paths to premature mortality considering the heterogeneity among old individuals.

The factors and paths identified in this study provide a valuable foundation for future research. These findings can guide future investigations on premature mortality using different datasets and inspire a more rigorous exploration of causal relationships. By building on this foundation, future studies can further contribute to a deeper understanding of the associations we observed.

## Electronic supplementary material

Below is the link to the electronic supplementary material.

Supplementary Material 1

## Data Availability

The datasets used and analyzed during the current study are available from the corresponding author upon reasonable request. The research was carried out in accordance with relevant guidelines and regulations.
