# Supplementary material for: Application of GUHA data mining method in cohort data to explore paths associated with premature death: a 29-year follow-up study
Source: BMC Med Res Methodol. 2025 Jan 27;25:20. doi: 10.1186/s12874-025-02477-6 (PMC11771032; doi:10.1186/s12874-025-02477-6)
Supplement: Supplementary file 1 — Supplementary Material 1 [file 12874_2025_2477_MOESM1_ESM.docx]

**Appendix**

Table A: Description of the factors (attributes) used in the analysis and percentage of the predicates.

| **Variable or question** | **Responses/ and responses modification** | **New categories for the responses** | **Percntage** | **Variable or question** | **Responses/ and responses modification** | **New categories for the responses** | **Percentage** |
| --- | --- | --- | --- | --- | --- | --- | --- |
| Age |  | (Median) | 78 |  |  |  |  |
| Gender | 1= female  2 = male |  | 55.2 | Working hours | 1-2 h  3-4 h  5-6 h  missing |  | 69.2  12.4  17.4  1.0 |
| Marital status | 1 = single  2 = married/co-habiting  3 = divorced/separated  4 = widowed  (1=1) (2=2) (3=1) (4=1) | 1 = Married  2 = Unmarried | 74.5 | What working hours do you have? | 1 = daytime job  2 = regular evening job  3 = regular night-time job  4 = two-shift work  5 = three-shift work  6 = other working hours  (1=1) (2=1) (3=2) (4=2) (5=3) (6=3) | 1= regular day or evening job  2 = regular night-time job or two-shifts work  3 = three-shift work or other working hours  missing | 69.2  12.4  17.4  1.0 |
| Social class | 31 = Secondary school teachers and senior officers  42 = Other lower officials  51 = Professional employees  52 = Unskilled employees |  | 10.5  43.7  25.0  20.7 | How many times a month do your working hours change? | 0 = 0  else =1 | 0 = no  1 = yes  not reported | 34.3  18.7  47.0 |
| Satisfaction with pay level | 1 = very satisfied  2 = quite satisfied  3 = hard to say  4 = quite unsatisfied  5 = very unsatisfied  missing |  | 3.1  30.0  17.0  24.2  12.7  13.0 | Did you work extra hours last week? | 0 = 0  else = 1 | 0 = no  1 = yes  missing | 74.5  21.8  3.7 |
| Years in full-time education | Elementary school or none  Primary school or part of secondary school  High school  University  missing |  | 18.8  46.7  14.2  8.5  11.8 | Have your working hours affected or restricted your family or other relationships? | 0=not at all  1=a little  2=to some extent  3 =a lot  (0=0) 0 (1= 0) (2=1) (3=1) | 0 = no  1 = yes  missing | 69.4  19.3  11.3 |
| Financial situation | 1 = very good  2 = quite good  3 = satisfactory  4 = quite poor  5 = very poor  (1=1) (2=1) (3=2) (4=3) (5=3) | 1 = good  2 = satisfactory  3 = poor  missing | 33.5  60.2  4.6  1.7 | Do your working hours affect your leisure activities? | 0 = not at all  1 = a little  2 = to some extent  3 =a lot  (0=0) (1=0) (2=1) (3=1) | 0 = no  1 = yes  missing | 63.5  23.4  13.1 |
| Pension security | 1 = very good  2 = pretty good  3 = moderate  4 = quite bad  5 = very bad  6 = I don’t know what it is like   (1=1) (2=1) (3=2) (4=3) (5=3) (6=4) | 1 = good  2 = satisfactory  3 = poor  4 = don’t know  missing | 32.8  42.7  9.4  13.6  21.23 | Do your working hours cause tiredness | 0 = not at all  1= a little  2 = to some extent  3 = a lot  (0=0) (1=0) (2=1) (3=1) | 0 = no  1 = yes  missing | 58.5  29.1  12.4 |
| Age at entry into paid employment | 1 thru 15=1  16 thru 30=2  Else=3 | 1 = under 15 y  2 = age16-30 y  3 = 31 or above  missing | 28.5  64.0  5.4  4.8 | Do your working hours cause nervousness? | 0 = not at all  1 = a little  2 = to some extent  3 = a lot  (0=0) (1=0) (2=1) (3=1) | 0 = no  1 = yes  missing | 71.1  11.8  17.1 |
| SRH: How would you rate your current health compared to your age peers | 1 = much better  2 = slightly better  3 = the same  4 = slightly worse  5 = much worse  (2=1) (1=1) (3=2) (4=3) (5=3) | 1 = good  2 = the same  3 = poor | 22.2  49.5  28.3 | Form of salary | 1 = monthly salary  2 = hourly wages  missing |  | 71.6  26.5  11.9 |
| Diseases affecting daily life | 1 = not at all  2 = quite little  3 = to some extent  4 = quite a lot  5 = very much  (0=0) (1=1) (2=1) (3=2) (4=2) (5=3) | 0 = not at all  1 = to some extent  2 = a lot    missing | 44.5  40.9  13.0   1.6 | To what extent do the following factors adversely affect your current work? / Detrimental factors at work: warm or cold working environment | 0 = not at all  1 = a little  2 = to some extent  3 = a lot  (0=0) (1=0) (2=1) (3=1) | 0 = no  1 = yes  missing | 50.2  41.6  8.2 |
| Diseases or injuries affecting work | 1 = not at all  2 = I can manage my job, but diseases or injuries cause symptoms.  3 = I sometimes have to pace myself at work, or change the way I work  4 = because of my condition, I think I could only manage part-time work  5 = I feel completely unable to work  (0 = 0) (2+3 =1) (4 =2) (5 =3) | 0 = none  1 = still work and sometimes have pace myself from work  2 = may be part-time work due to my conditions  3= feels unable to work | 44.5  40.5  12      2.9 | Detrimental factors at work:  Dry or humid work environment | 0 = not at all  1 = a little  2 = to some extent  3 = a lot  (0=0) (1=0) (2=1) (3=1) | 0 = no  1 = yes  missing | 61.8  23.7  14.5 |
| Changed jobs due to illness | 0 = never  1 = yes, over 5 years ago  2 = yes, during the last 5 years  (0=0) (1=1) (2=1) | 0 = no  1 = yes | 92.8  7.2 | Detrimental factors at work:  Dirty work environment | 0 = not at all  1 = a little  2 = to some extent  3 = a lot  (0=0) (1=0) (2=1) (3=1) | 0 = no  1 = yes  missing | 62.1  23.4  14.5 |
| Symptoms that impair functioning:  -chest pain that slows walking  -shortness of breath that slows walking  -back pain that interferes with dressing  -other symptom that interferes with performance | 0 = no  1 = sometimes  2 = usually  (0=0) (1=1) (2=1) | 0 = no  1 = yes" | 32.2  67.8 | Detrimental factors at work:  pollution,  dust, smoke, steam | 0 = not at all  1 = a little  2 = to some extent  3 = a lot  (0=0) (1=0) (2=1) (3=1) | 0 = no  1 = yes  missing | 58.3  28.9  12.8 |
| Musculoskeletal pain  Pain in  neck, shoulders elbow, wrist, fingers, lower back, thighs, and ankle or foot | 0 = no  1 = sometimes  2 = usually  (0=0) (1=1) (2=1) | 0 = no  1 = yes | 14.8  85.5 | Detrimental factors at work:  acids, bases, solvents | 0 = not at all  1 = a little  2 = to some extent  3 = a lot  (0=0) (1=0) (2=1) (3=1) | 0 = no  1 = yes  missing | 58.3  28.9  12.8 |
| Pains or aches that interferes with work | 0 = not at all  1 = I can manage my job, although the pain/ache bothers me a little  2 = sometimes I have to pace myself or change my way of working  3 = I often have to pace myself or change my way of working.  4 = because of my condition, I think I could only manage part-time work  5 = I feel completely unable to work | 0 = none  1 = a little or sometimes have  2 = pain that would require adjustments to work  3 = feels unable to work | 17.2  66.8  12.9    3.2 | Detrimental factors at work:  noise from machinery or equipment | 0 = not at all  1 = little  2 = to some extent  3 = a lot  (0=0) (1=0) (2=1) (3=1) | 0 = no  1 = yes  missing | 63.7  22.2  14.1 |
| Absences from work for health reasons | 0  1 -9 times  (0=0) (1=1) (2=2) (3-9=2) | 0 = no  1 = 1 times  2 = 2 times  3 = 3-9 times | 66.1  24.9  6.2  2.9 | Detrimental factors at work:  restless working environment | 0 = not at all  1 = little  2 = to some extent  3 = a lot  (0=0) (1=0) (2=1) (3=1) | 0 = no  1 = yes  missing | 62.4  24.9  12.7 |
| Work ability score | (0=0) (1=0) (2=0) (3=0) (4=0) (5=1) (6=1) (7=2) (8=2) (9=2) (10=2) | 0 = poor  1 = medium  2 = good | 6.6  1.7  76.7 | In terms of mental requirements, is your current job…? / Mental requirements of work | 1 = too light  2 = a bit too light  3 = appropriate  4 = a bit too hard  5 = too hard  (1=1) (2=1) (3=2) (4=3) (5=3) | 1 = too light  2 = appropriate  3 = too heavy  missing | 2.9  55.2  35.5  6.4 |
| Comparing work ability to two years ago | 1 = much better  2 = slightly what better  3 = similarly  4 = slightly worse  5 = much worse  (1=1) (2=1) (3=2) (4=3) (5=3) | 1 = worse  2 = same  3 = better  missing | 4.8  54.4  40.2  0.6 | In terms of physical requirements, is your current job…?  / Physical requirements of work | 1 = too light  2 = a bit too light  3 = appropriate  4 = a bit too hard  5 = too hard  (1=1) (2=1) (3=2) (4=3) (5=3) | 1 = too light  2 = appropriate  3 = too heavy  missing | 4.8  52.8  38.7  3.7 |
| How would you rate your ability to work in relation to the physical demands of your job? / Physical work ability | 1= very good  2 = quite good  3 = moderate  4 = quite poor  5 = very poor  (1=1) (2=1) (3= 2) (4= 3) (5= 3) | 1 = good  2 = moderate  3 = poor  missing | 35.2  49.1  14.9  3.5 | “Does your work require …”  repetitive movements | 0 = not at all/very seldom  1 = seldom  2 = moderately  3 = often  4 = very often  (0=0) (1=0) (2=1) (3=2) (4=2) | 0 = no  1 = yes, moderately  2 = yes, often  missing | 29.8  20.2  43.5  6.5 |
| How would you rate your ability to work in relation to the mental demands of your job? / Mental work ability | 1 = very good  2 = quite good  3 = moderate  4 = quite poor  5 = very poor  (1=1) (2=1) (3= 2) (4= 3) (5= 3) | 1 = good  2 = moderate  3 = poor  missing | 51.0  41.9  5.8  1.3 | “Does your work require …”  standing still at work | 0 = not at all/very seldom  1 = seldom  2 = moderately   3=often  4 = very often  (0=0) (1=0) (2=1) (3=2) (4=2) | 0 = no  1 = yes, moderately  2 = yes, often  missing | 58.1  17.0  15.2  9.7 |
| Work ability index score | 7 thru 27 = 1  28 thru 36 = 2  37 thru 43 = 3  44 thru 49 = 4 | (7 thru 27=1) = poor  (28 thru 36=2) = moderate  (37 thru 43=3) = good  (44 thru 49=4) = excellent  missing | 11.7  27.9  30.6  13.6  16.2 | “Does your work require …”  awkward postures at work | 0 = not at all/very seldom  1 = seldom  2 = moderately  3 = often  4 = very often  (0=0) (1=0) (2=1) (3=2) (4=2) | 0 = no  1 = yes, moderately  2 = yes, often  missing | 35.5  19.7  42.0  2.8 |
| Number of diagnosed diseases | 1-7 diseases  (1=1) (2=1) (3-7= 2) | 1 = less than 3  2 = 3 or more than 3 diseases | 19.4  80.5 | “Does your work require …”  moving or walking a lot at work | 0 = not at all/very seldom  1 = seldom  2 = moderately  3 = often  4 =very often  (0=0) (1=0) (2=1) (3=2) (4=2) | 0 = no  1 = yes, moderately  2 = yes, often  missing | 26.3  22.8  45.3  5.6 |
| Is it easy for you to fall asleep?  Sleeping difficulties | 1 = very easy  2 = quite easy  3 = not easy nor difficult  4 = quite difficult  5 = very difficult  (1= 1) (2=1) (3=2) (4=3) (5= 3) | 1 = easy  2 = not very easy  and not very difficult  3 = difficult  missing | 19.8  32.9  30.9  11.4 | “Does your work require …”  Carrying and lifting heavy objects at work | 0 = not at all/very seldom  1 = seldom  2 = moderately  3 = often  4 = very often  (0=0) (1=0) (2=1) (3=2) (4=2) | 0 = no  1 = yes, moderately  2 = yes, often  missing | 34.8  17.9  41.5  5.8 |
| Do you feel anxious? | 1 = never  2 = quite seldom  3 = sometimes  4 = quite often  5 = always or very often  (0=1) (1=1) (2= 2) (3=3) (4= 3) | 1 = never or seldom  2 = sometimes  3 = often  missing | 33.1  43.0  12.4  11.5 | “Does your work require …”  Sitting in the same place at work | 0 = not at all/very seldom  1 = seldom  2 = moderately  3 = often  4 = very often  (0=0) (1=0) (2=1) (3=2) (4=2) | 0 = no  1 = yes, moderately  2 = yes, often  missing | 53.7  15.7  21.7  8.9 |
| Have you recently been able to enjoy your daily activities? | 4 = often  3 = quite often  2 = sometimes  1 = quite seldom  0 = never  (0=1) (1=1) (2=2) (3=3) (4=3) | 1 = never  2 = sometimes  3 = often  missing | 11.8  28.3  46.8  13.1 | “Does your work require …”  Interaction at work | 0 = not at all/very seldom  1 = seldom  2 = moderately  3 = often  4 = very often  (0=0) (1=0) (2=1) (3=2) (4=2) | 0 = no  1 = yes, moderately  2 = yes, often | 13.7  23.7  62.6 |
| Do you feel tightness, pain or anxiety in your chest? | 0 = never  1 = quite seldom  2 = sometimes  3 = quite often  4 = often or almost always  (0=1) (1=1) (2=2) (3=3) (4=3) | 1 = never  2 = sometimes  3 = often  missing | 57.3  22.5  7.1  13.1 | “Does your work require …”  Responsibility over others at work | 0 = not at all/very seldom  1 = seldom  2 = moderately  3 = often  4 = very often  (0=0) (1=0) (2=1) (3=2) (4=2) | 0 = no  1 = yes, moderately  2 = yes, often  missing | 19.6  13.1  61.2  6.1 |
| When you go to work, do you feel you would rather stay at home? | 0 = never  1 = quite seldom  2 = sometimes  3 = quite often  4 = often or almost always  (0=1) (1=1) (2=2) (3=3) (4=3) | 1 = never  2 = sometimes  3 = often  missing | 52.1  25.6  10.6  11.7 | To what extent do the following interrupt your work?  work responsibility | 0 = not at all  1 = a little  2 = to some extent  3 = a lot  (0=0) (1=0) (2=1) (3=2) | 0 = no  1 = yes  2 = a lot  missing | 57.3  23.8  11.6  7.3 |
| Remembering requires effort | 0 = never  1 = quite seldom  2 = sometimes  3 = quite often  4 = often or almost always  (0=1) (1=1) (2=2) (3=3) (4=3) | 1 = never  2 = sometimes  3 = often  missing | 23.2  43.8  21.5  11.5 | To what extent do the following interrupt your work?  Time pressure | 0 = not at all  1 = a little  2 = to some extent  3 = a lot  (0=0) (1=0) (2=1) (3=2) | 0 = no  1 = yes  2 = a lot  missing | 42.0  30.6  21.4  6 |
| Feeling nervous | 0 = never  1 = quite seldom  2 = sometimes  3 = quite often  4 = often or almost always  (0=1) (1=1) (2=2) (3=3) (4=3) | 1 = never  2 = sometimes  3 = often  missing | 40.9  39.2  8.6  11.3 | To what extent do the following interrupt your work?  Awkward working postures | 0 = not at all  1 = a little  2 = to some extent  3 = a lot  (0=0) (1=0) (2=1) (3=2) | 0 = no  1 = yes  2 = a lot  missing | 50.1  26.9  16.5  6.5 |
| Feeling dizzy | 0 = never  1 = quite seldom  2 = sometimes  3 = quite often  4 = often or almost always  (0=1) (1=1) (2=2) (3=3) (4=3) | 1 = never  2 = sometimes  3 = often  missing | 60.9  20.9  6.0  12.2 | To what extent do the following interrupt your work?  Being overly controlled at work | 0 = not at all  1 = a little  2 = to some extent  3 = a lot  (0=0) (1=0) (2=1) (3=2) | 0 = no  1 = yes  2 = a lot | 82.0  7.7  2.3 |
| Feeling reluctant | 0 = never  1 = quite seldom  2 = sometimes  3 = quite often  4 = often or almost always  (0=1) (1=1) (2=2) (3=3) (4=3) | 1 = never  2 = sometimes  3 = often  missing | 50.9  34.2  13.8  1.1 | To what extent do the following interrupt your work?”  Forced work pace | 0 = not at all  1 = a little  2 = to some extent  3 = a lot  (0=0) (1=0) (2=1) (3=2) | 0 = no  1 = yes  2 = a lot  missing | 65.7  16.6  10.2  7.5 |
| Is your continuity of sleep disrupted?  Sleep quality | 0 = I usually do not wake up in the night  1 = I wake up once a night  2 = I wake up a couple of times a night  3 = I wake up 3-4 times a night  4 = I sleep intermittently  (0=1) (1=1) (2=2) (3=3) (4=3) | 1 = “none or very few” good quality  2 = “some” sleep disturbance  3 = “quite a lot of lot” disturbance  missing | 56.2  26.4  16.6  0.8 | To what extent do the following interrupt your work?  Isolation or loneliness at work | 0 = not at all  1 = a little  2 = to some extent  3 = a lot  (0=0) (1=0) (2=1) (3=2) | 0 = no  1 = yes  2 = a lot  missing | 84.6  5.4  1.9  8.1 |
| Do you keep losing your thoughts? | 0 = never  1 = quite seldom  2 = sometimes  3 = quite often  4 = often or almost always  (0=1) (1=1) (2=2) (3=3) (4=3) | 1 = never  2 = sometimes  3 = often  missing | 60.0  31.6  6.6  1.8 | In your current job, to what extent can you influence your work environment? | 0 = not at all  1 = a little  2 = to some extend  3 = a lot  (0=0) (1=0) (2=1) (3=2) | 0 = no  1 = yes  2 = a lot  missing | 51.7  29.4  12.2  6.7 |
| Do you ever feel like you want to skip work for no compelling reason? | 0 = never  1 = quite seldom  2 = sometimes  3 = quite often  4 = often or almost always  (0=1) (1=1) (2=2) (3=3) (4=3) | 1 = never  2 = sometimes  3 = often  missing | 78.7  14.7  4.9  1.7 | “If you could change your job but be paid the same, would you do that? | 1 = I wouldn’t change  2 = I cannot say I would change  3 = I would change to the same occupation  4 = I would switch to another occupation  missing |  | 37.9  30.5  8.9  9.9  12.8 |
| Have you felt depressed? | 0 = never  1 = quite seldom  2 = sometimes  3 = quite often  4 = often or almost always  (0=1) (1=1) (2=2) (3=3) (4=3) | 1 = never  2 = sometimes  3 = often  missing | 61.7  29.7  7.4  1.2 | Alcohol use | 1 = never  2 = not so often  3 = once a month  4 = couple of times a month  5 = once a week  6 = couple of times a week  7 = daily  (1= 1) (2=2) (3=3) (4=4) (5= 4) (6=5) (7=5) | 1 = never  2 = not so often  3 = once a month  4 = couple of times a month or once a week  5 = couple of times a week or daily  missing | 38.5  9.1  11.1  10.5    0.1  30.7 |
| Have you felt active and energetic lately? | 0 = never  1 = quite seldom  2 = sometimes  3 = quite often  4 = often or almost always  (0=1) (1=1) (2=2) (3=3) (4=3) | 1 = never  2 = sometimes  3 = often  missing | 12.6  29.9  56.0  1.5 | How often do you exercise? / Leisure-time physical activity | 1 = brisk exercise at least two times a week  2 = brisk exercise at least once a week  3 = some exercise once a week  4 = some less than once a week  5 = I do not exercise  missing |  | 3.7  17.0  36.1  27.6  0.0  15.6 |
| Have you recently felt hopeful for the future? | 0 = never  1 = quite seldom  2 = sometimes  3 = quite often  4 = often or almost always  (0=1) (1=1) (2=2) (3=3) (4=3) | 1 = never  2 = sometimes  3 = often | 27.7  28.1  42.3 | How would you describe is your physical fitness compared to people of the same age?  / self-rated physical fitness | 1 = much better  2 = is somewhat better  3 = similar  4 = is even worse  5 = much worse  (1=1) (2=1) (3=2) (4=3) (5=3) | 1 = better  2 = same  3 = worse  missing | 24.1  49.8  24.2  1.9 |
| To what extent do you enjoy your work? | 1 = very well  2 = good  3 = moderate  4 = bad  5 = very badly  (4=1) (5=1) (3=2) (1=3) (2=3) | 1 = very little  2 = sometimes  3 = very much | 2.9  22.5  73.9 | Are you happy with your current life situation? | 1 = very satisfied  2 = quite satisfied  3 = hard to say  4 = quite unsatisfied  5 = very unsatisfied  (1=1) (2=1) (3=2) (4=3) (5=3) | 1 = yes, satisfied  2 = don’t know  3 = unsatisfied  missing | 78.8  14.4  5.1  1.7 |
| Has your workload in the past two years …  recently…? | 1 = decreased substantially  2 = decreased slightly  3 = stayed the same  4 = increased slightly  5 = increased extensively  (1=3) (2=3) (3=2) (4=1) (5=1) | 1 = increased  2 = stayed the same  3 = decreased | 41.4  48.1  8.4 | Ever smoked previously | (0=0)  (else=1) | 0 = no  1 = yes  missing | 51.9  44.3  3.8 |
| Tobacco index | 0=not  1=less than 20 cigarettes  2=more than 20 cigarettes |  | 80.6  10.0  9.4 | Duration of commute to work | 1 = less than 20 min  2 = 15 to 29 min  3 = 30 -45 min  4 = more than 45 min |  | 34.3  40.1  20.9  4.7 |
| How important are the following reasons when one considers the timing of retirement?   mental strain | 0 = not important at all  1 = somewhat important  2 = quite important  3 = very important  (0=1) (1=1) (2=2) (3=2) | 1 = no  2 = yes | 5.1  90.8 | How would you describe your household’s standard of living?” | 1 = very good  2 = quite good  3 = satisfactory  4 = quite poor  5 = very poor  (1=1) (2=1) (3=2) (4=3) (5=3) | 1 = good  2 = satisfactory  3 = poor  missing | 56.4  39.5  2.7  1.4 |
| How important are the following reasons when one considers the timing of retirement?  physical work strain | 0 = not important at all  1 = somewhat important  2 = quite important  3 = very important  (0=1) (1=1) (2=2) (3=2) | 1 = no  2 = yes | 2.0  95.1 | Current health for being able to continue to work for two years | 0 = I will be retired in two years  1 = quite sure  2 = I am not sure  3 = hardly  (3=3) (2=2) (1=1) (0=2) | 1 = sure  2 = I am not sure  3 = hardly  missing | 50.9  44.4  3.8  0.8 |
| How important are the following reasons when one considers the timing of retirement?  reduced workability due to disease | 0 = not important at all  1 = somewhat important  2 = quite important  3 = very important  (0=1) (1=1) (2=2) (3=2) | 1=no  2=yes | 0.8  99.2 | How important are the following reasons when one considers the timing of retirement?  Changes in the content of work tasks | 0 = not important at all  1 = somewhat important  2 = quite important  3 = very important  (0=1) (1=1) (2=2) (3=2) | 1 = no  2 = yes  missing | 35.4  57.0  7.6 |
| How important are the following reasons when one considers the timing of retirement?  gender | 0 = not important at all  1 = somewhat important  2 = quite important  3 = very important  (0=1) (1=1) (2=2) (3=2) | 1=no  2=yes  missing | 67.5  27.0  5.5 | BMI | Calculated using self-reported height and weight and categorized as | BMI<19.00= underweight  19≥BMI≤24.99= healthy overweight ≥25  missing | 0.7  45.4  42.0  11.9 |
| How important are the following reasons when one considers the timing of retirement?  employment situation | 0 = not important at all  1 = somewhat important  2 = quite important  3 = very important  (0=1) (1=1) (2=2) (3=2) | 1 = no  2 = yes  missing | 27.3  67.5  5.2 | Medication use | Any medication for pain, heart diseases, Blood pressure, asthma and stomach pain, and sedative or sleeping pills | 0 = no  1 = yes | 31.5  68.4 |

**Appendix B.** Paths to Premature Death and Posterior Distributions Related to Premature Death. Each path is a combination of predicates significantly associated with premature death identified across six tasks. Each task has different *Base* and *p* values for the total study. The Histogram of Samples from the posterior distribution of the *above-average* parameters for the contingency table of each path. The histograms illustrate samples from the posterior distribution of above-average parameters for the contingency table of each path. For instance, the first histogram shows a 99% certainty that premature death is at least 1.449 times more prevalent among individuals who meet the following criteria: Do not have musculoskeletal diseases, smoked previously, have symptoms impairing daily functioning, work only 1–2 hours per day, and have the intension to retire due to reduced work ability due to diseases^[[1]](#footnote-2)^.

These visualizations demonstrate tightly clustered distributions around the mean, indicating high precision in estimating the parameters of interest.

**TASK 1**. **BASE = 400, p = 0.55. With p-value in between 0.552 – 0.568 and a-value in between 409 – 440, the following 7 Paths are found**:

No musculoskeletal diseases & Smoked previously & Having symptoms that impair functioning & Working hours is 1 or 2 hours & Intention to retire: reduced work ability due to diseases


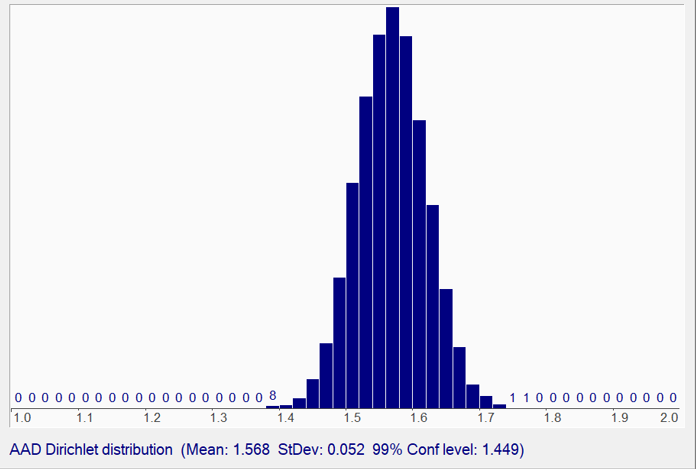


No musculoskeletal diseases & Having pain & Smoked previously & Having symptoms that impair functioning & Working hours is 1 or 2 hours


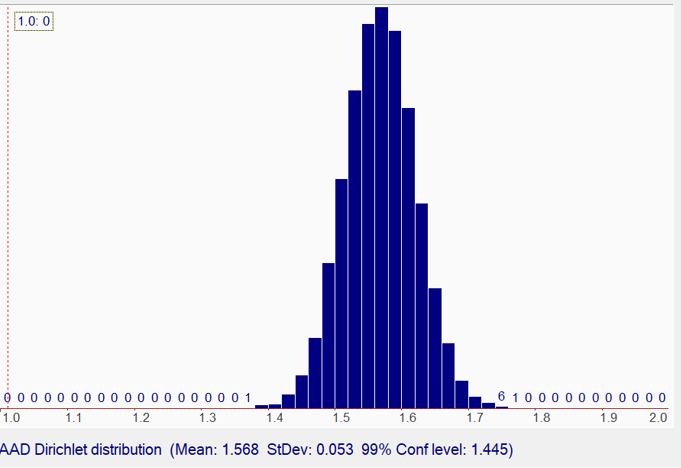


No musculoskeletal diseases & Having pain & Smoked previously & Having symptoms that impair functioning & male sex


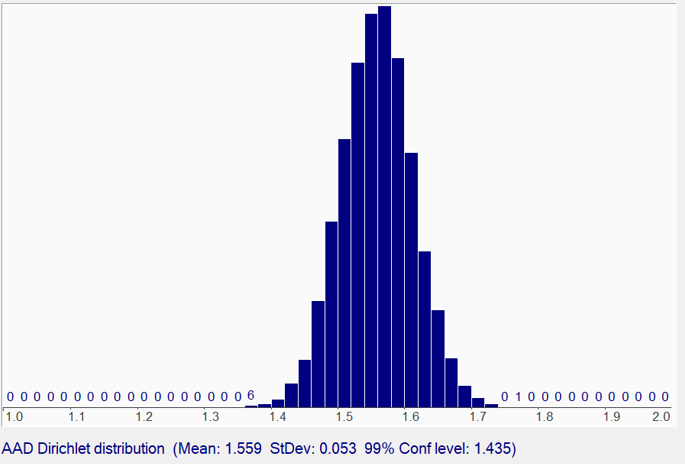


Having pain & Smoked previously & Having symptoms that impair functioning & Working hours is 1 or 2 hours & Intention to retire: reduced work ability due to diseases


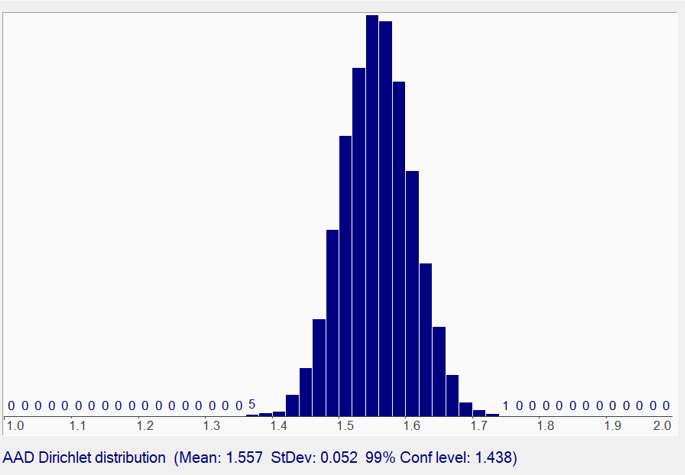


No musculoskeletal diseases & Intention to retire: physical work strain & Smoked previously & Having symptoms that impair functioning & Working hours is 1 or 2 hours


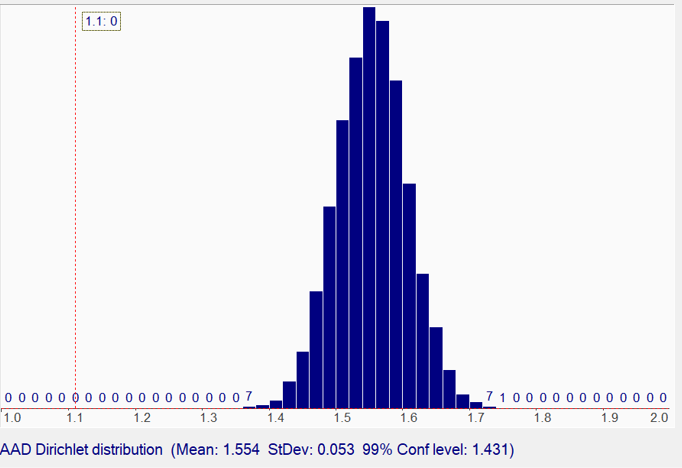


Smoked previously & Having symptoms that impair functioning & Working hours is 1 or 2 hours & Intention to retire: reduced work ability due to diseases


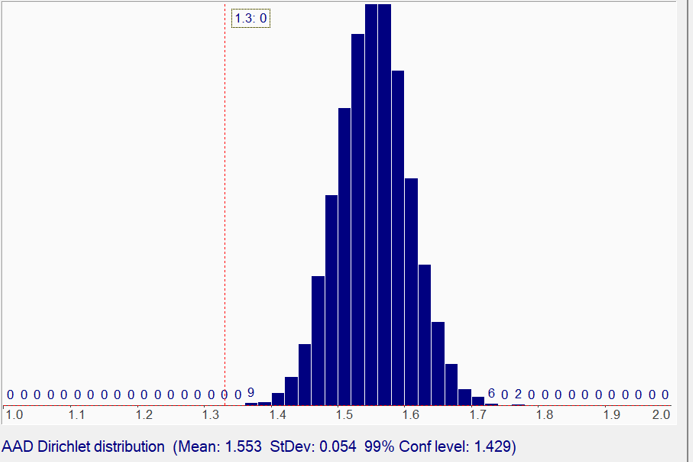


No musculoskeletal diseases & Smoked previously & Having symptoms that impair functioning & Male sex & Intention to retire: reduced work ability due to diseases


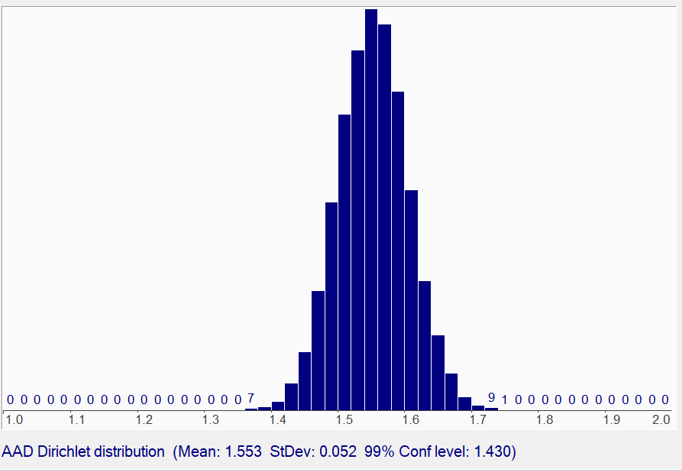


**TASK 2. BASE = 300, p = 0.68. With p-value in between 0.68 – 0.784 and a-value in between 300 – 322, the following 11 Paths are found:**

Having pain & Smoked previously & Poor self-rated health & Having symptoms that impair functioning & Use of medication


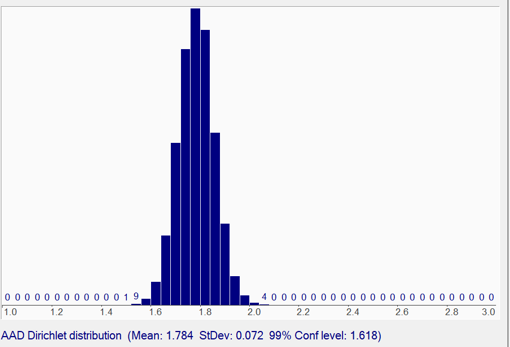


Smoked previously & Poor self-rated health & Having symptoms that impair functioning & Use of medication & Intention to retire: reduced work ability due to diseases


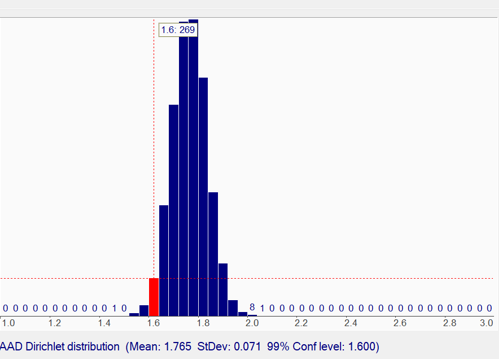


No musculoskeletal diseases & Having pain & Smoked previously & Poor self-rated health & Having symptoms that impair functioning


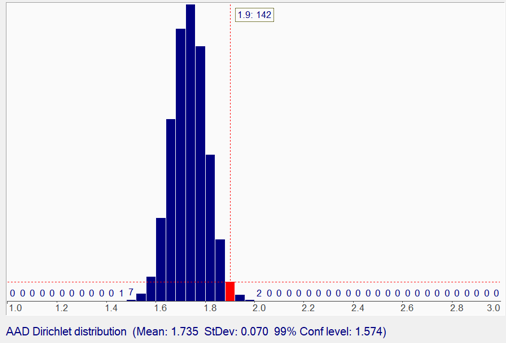


Having pain & Intention to retire: physical work strain & Smoked previously & Poor self-rated health & Having symptoms that impair functioning


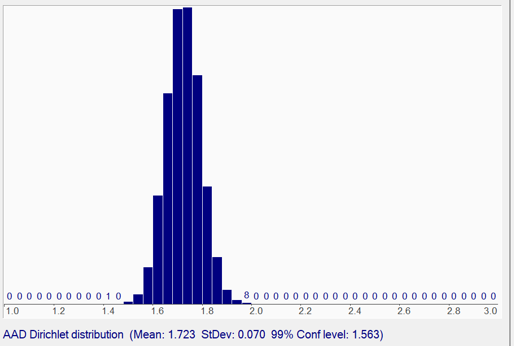


No musculoskeletal diseases & Smoked previously & Poor self-rated health & Having symptoms that impair functioning & Intention to retire: reduced work ability due to diseases


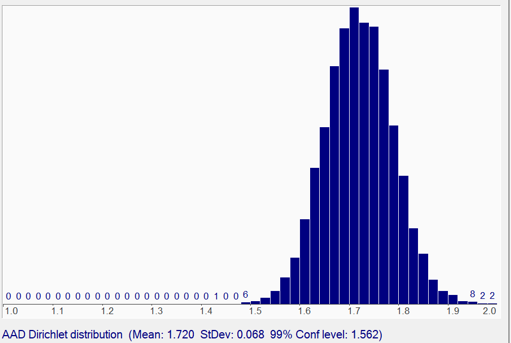


Having pain & Smoked previously & Poor self-rated health & Having symptoms that impair functioning & Intention to retire: reduced work ability due to diseases


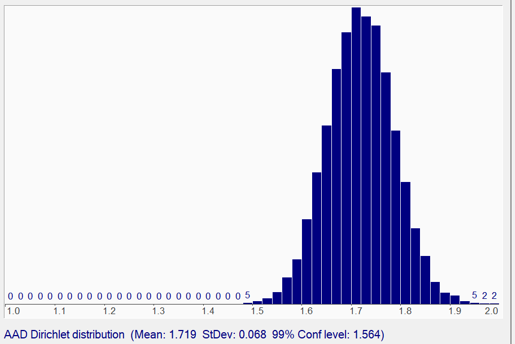


Having pain & Smoked previously & Poor self-rated health & Use of medication & Intention to retire: reduced work ability due to diseases


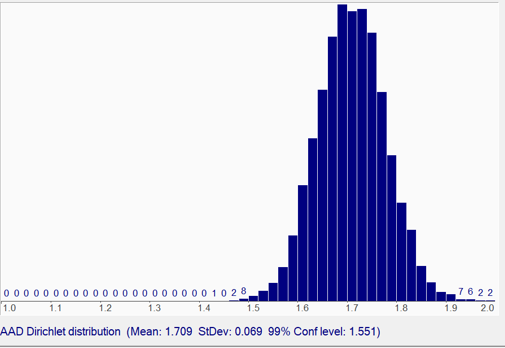


Intention to retire: physical work strain & Smoked previously & Poor self-rated health & Having symptoms that impair functioning & Intention to retire: reduced work ability due to diseases


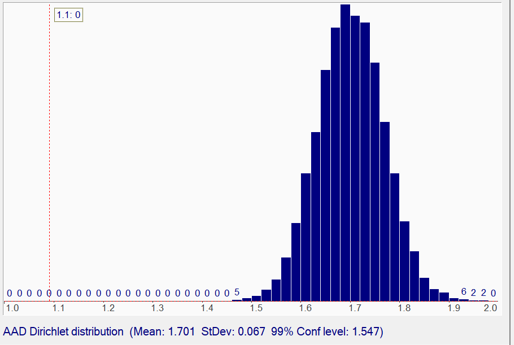


No musculoskeletal diseases & Smoked previously & Poor self-rated health & Use of medication & Intention to retire: reduced work ability due to diseases


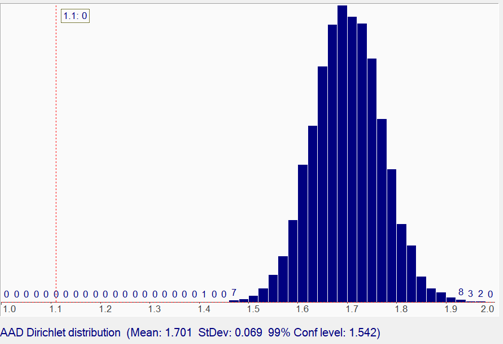


Intention to retire: physical work strain & Smoked previously & Poor self-rated health & Use of medication & Intention to retire: reduced work ability due to diseases


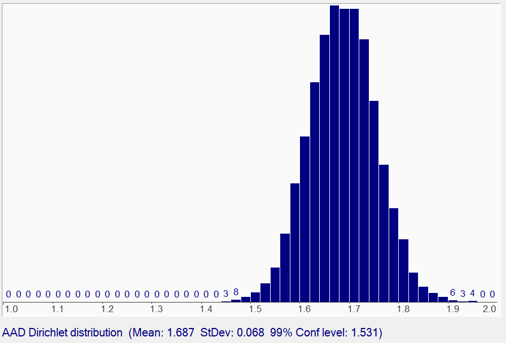


No musculoskeletal diseases & Having pain & Smoked previously & Poor self-rated health & Intention to retire: reduced work ability due to diseases


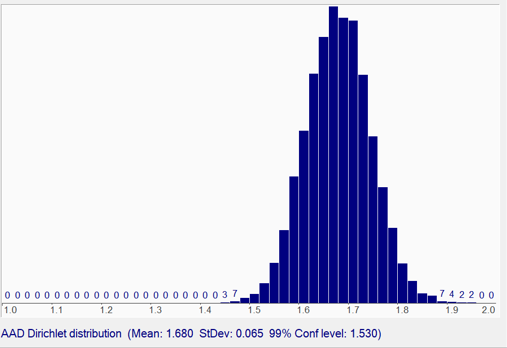


**TASK 3**. **BASE = 200, p = 1. With p-value in between 1.008 – 1.104 and a-value in between 202 – 210, the following 7 Paths are found:**

Smoking more than 20 cigarettes & No musculoskeletal diseases & Intention to retire: physical work strain & Smoked previously & Working hours is 1 or 2 hours


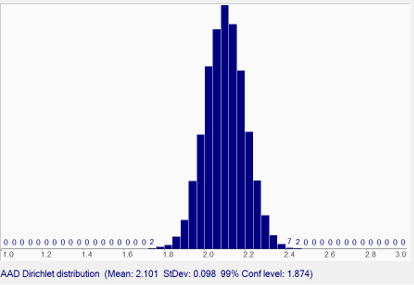


Smoking more than 20 cigarettes & No musculoskeletal diseases & Smoked previously & Working hours is 1 or 2 hours & Intention to retire: reduced work ability due to diseases


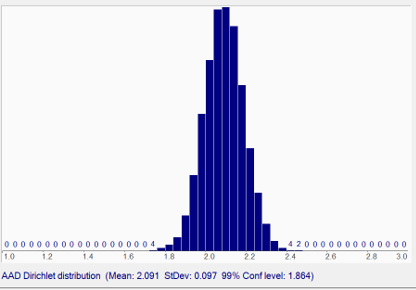


Smoking more than 20 cigarettes &Intention to retire: physical work strain & Smoked previously & Working hours is 1 or 2 hours & Intention to retire: reduced work ability due to diseases


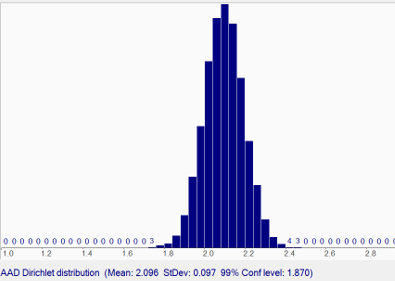


Smoking more than 20 cigarettes & No musculoskeletal diseases &Intention to retire: physical work strain & Working hours is 1 or 2 hours & Intention to retire: reduced work ability due to diseases


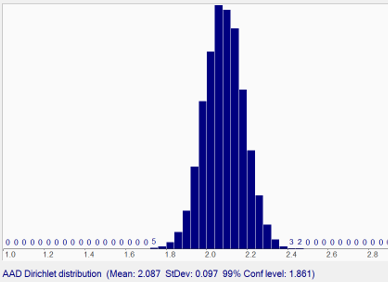


Smoking more than 20 cigarettes & Not changing work due to diseases & No musculoskeletal diseases & Smoked previously & Intention to retire: reduced work ability due to diseases


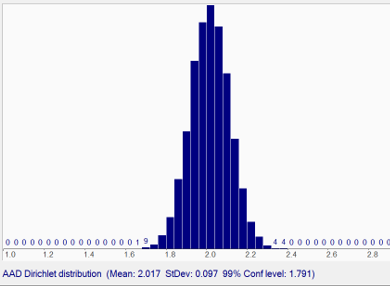


Smoking more than 20 cigarettes & Intention to retire: mental strain at work & No musculoskeletal diseases & Having pain & Smoked previously


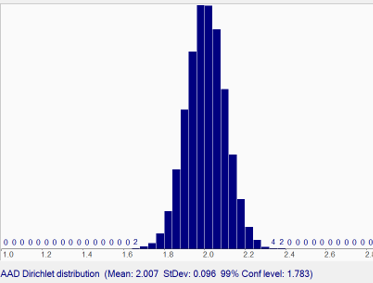


Smoking more than 20 cigarettes & Intention to retire: mental strain at work & No musculoskeletal diseases & Smoked previously & Male sex


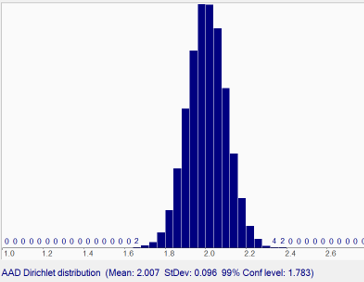


**TASK 4**. **BASE = 150, p =1.2. With p-value in between 1.2 – 1.207 and a-value in between 150 – 170, the following 6 Paths are found**:

Having symptoms that impair functioning & Working hours is 1 or 2 hours & Smoked previously & Smoking more than 20 cigarettes & No musculoskeletal diseases


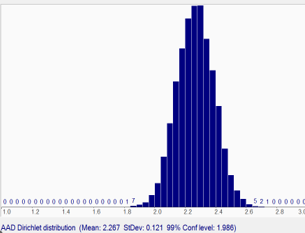


Having symptoms that impair functioning & Working hours is 1 or 2 hours & Intention to retire: reduced work ability due to diseases & Smoked previously & Smoking more than 20 cigarettes


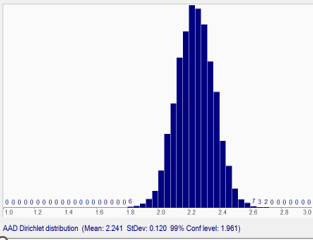


Having pain & Having symptoms that impair functioning & Smoked previously & Smoking more than 20 cigarettes & No musculoskeletal diseases


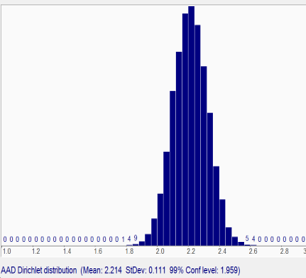


Not changing work due to diseases & Having pain & Working hours is 1 or 2 hours & Smoked previously & Smoking more than 20 cigarettes


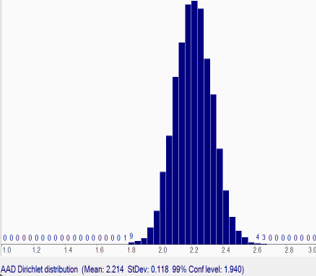


Intention to retire: mental strain at work & Having pain & Having symptoms that impair functioning & smoking more than 20 cigarettes & No musculoskeletal diseases


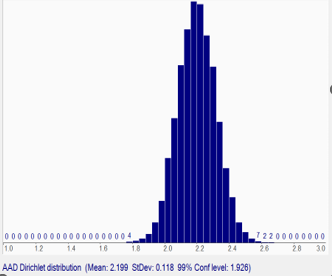


Having pain & Having symptoms that impair functioning & Male sex & Smoked previously & smoking more than 20 cigarettes


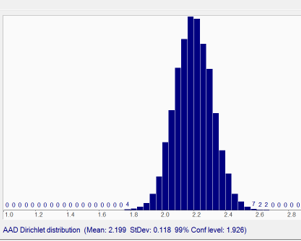


**TASK 5**. **BASE = 100, p =1.4. With p-value in between 1.401 – 1.525 and a-value in between 100 – 125, the following 21 Paths are found:**

Not changing work due to diseases & Having symptoms that impair functioning & Working hours is 1 or 2 hours & Use of medication & Smoking more than 20 cigarettes


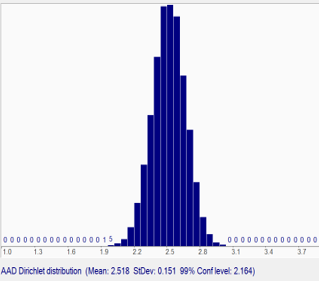


Married & Satisfied with life & Having symptoms that impair functioning & smoking more than 20 cigarettes & No musculoskeletal diseases


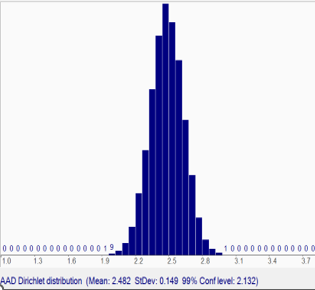


Married & Satisfied with life & Having symptoms that impair functioning & Smoked previously & Smoking more than 20 cigarettes


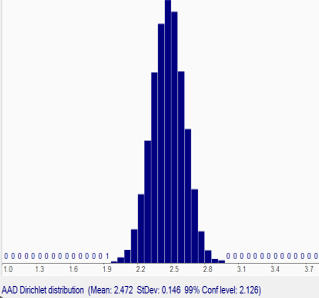


Married & Having pain & Satisfied with life& Having symptoms that impair functioning & Smoking more than 20 cigarettes


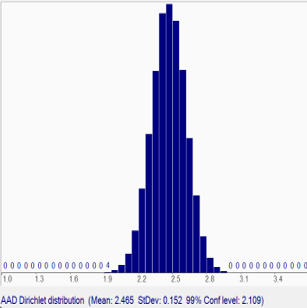


Not changing work due to diseases & Having symptoms that impair functioning & Working hours is 1 or 2 hours & Smoked previously & Smoking more than 20 cigarettes


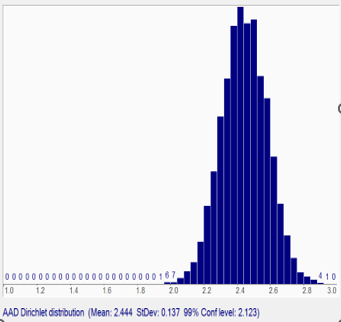


Having pain & Worse fitness compared to others at the same age& Smoked previously & Smoking more than 20 cigarettes & No musculoskeletal diseases


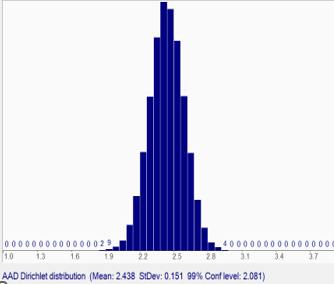


Poor self-rated health & Use of medication & Smoked previously & Smoking more than 20 cigarettes & No musculoskeletal diseases


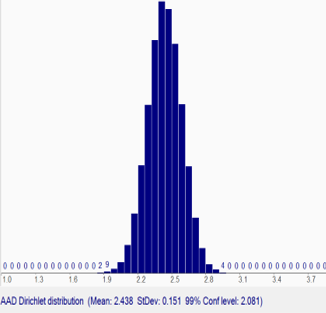


Poor self-rated health & Having symptoms that impair functioning & Use of medication & Smoked previously & Smoking more than 20 cigarettes


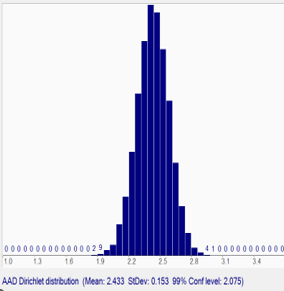


Satisfied with life & Having symptoms that impair functioning & Working hours is 1 or 2 hours & Smoked previously & Smoking more than 20 cigarettes


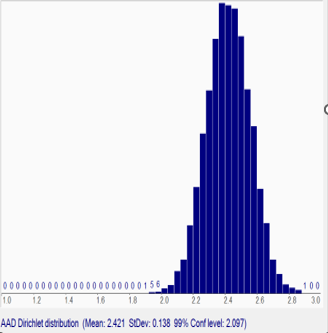


Not changing work due to diseases & Having symptoms that impair functioning & Working hours is 1 or 2 hours & Smoking more than 20 cigarettes & No musculoskeletal diseases


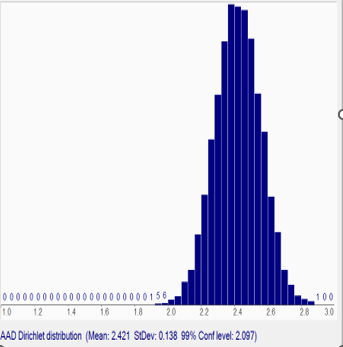


Not changing work due to diseases & Having pain & Having symptoms that impair functioning & Working hours is 1 or 2 hours & Smoking more than 20 cigarettes


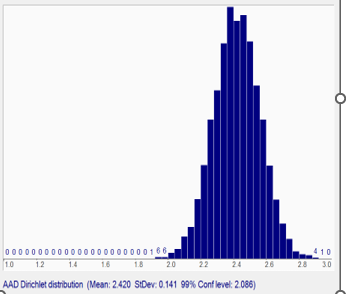


Married & Satisfied with life & Having symptoms that impair functioning & Intention to retire: reduced work ability due to diseases & Smoking more than 20 cigarettes


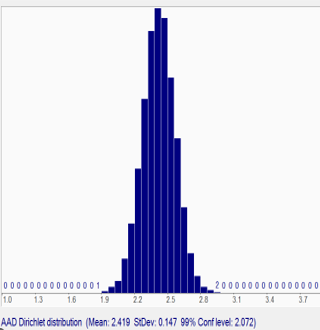


Having pain & Poor self-rated health & Use of medication & Smoked previously & Smoking more than 20 cigarettes


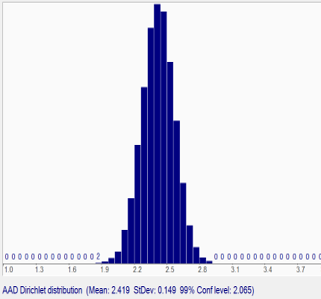


Satisfied with life& Having symptoms that impair functioning & Working hours is 1 or 2 hours & Smoking more than 20 cigarettes & No musculoskeletal diseases


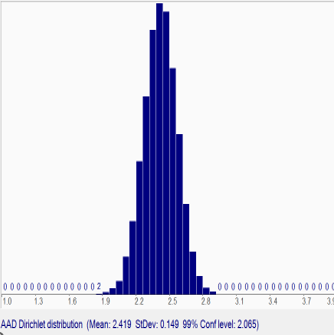


Intention to retire: physical work strain & Satisfied with life & Having symptoms that impair functioning & Working hours is 1 or 2 hours & Smoking more than 20 cigarettes


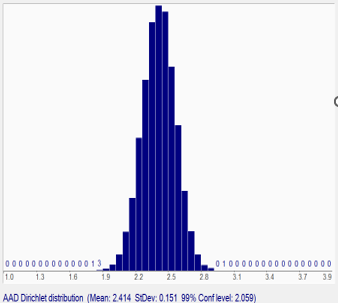


Married &Intention to retire: physical work strain & Satisfied with life & Having symptoms that impair functioning & Smoking more than 20 cigarettes


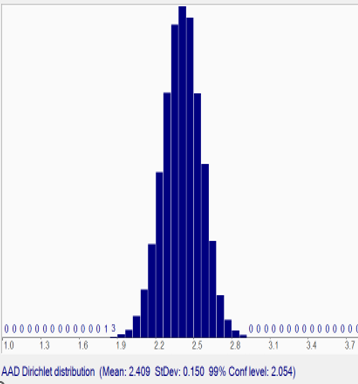


Married & Satisfied with life & Having symptoms that impair functioning & Male sex & Smoking more than 20 cigarettes


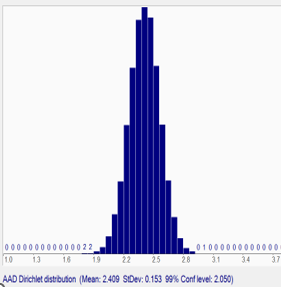


Not changing work due to diseases & Having symptoms that impair functioning & Use of medication & Smoking more than 20 cigarettes & No musculoskeletal diseases


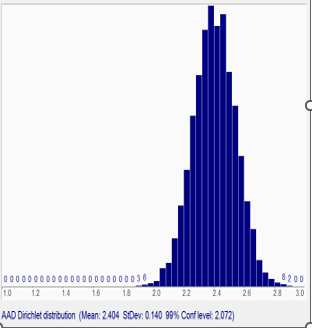


Having pain & Poor self-rated health & Male sex & Smoking more than 20 cigarettes & No musculoskeletal diseases


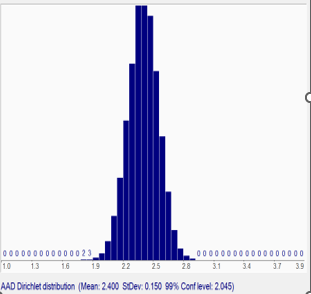


Satisfied with life & Having symptoms that impair functioning & Working hours is 1 or 2 hours & Intention to retire: reduced work ability due to diseases & Smoking more than 20 cigarettes


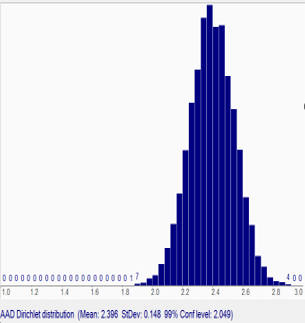


**TASK 6**. **BASE = 75, p =1.5. With p-value in between 1.5 – 1.599 and a-value in between 75 – 104, the following 15 Paths are found:**

Smoking more than 20 cigarettes & Not changing work due to diseases & Having pain & Poor self-rated health & Having symptoms that impair functioning


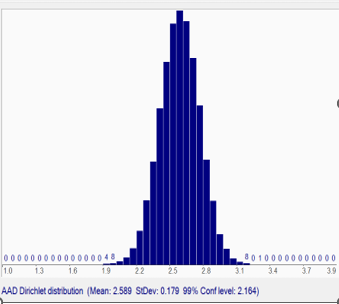


Smoking more than 20 cigarettes & Not changing work due to diseases & No musculoskeletal diseases & Poor self-rated health   & Use of medication


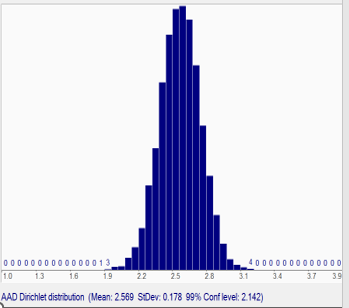


Smoking more than 20 cigarettes & Not changing work due to diseases & No musculoskeletal diseases & Poor self-rated health & Having symptoms that impair functioning


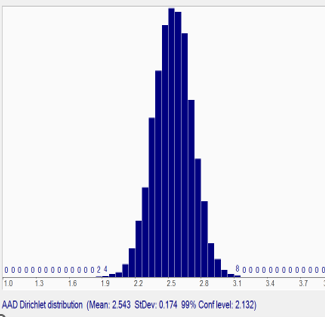


Smoking more than 20 cigarettes & Not changing work due to diseases & Smoked previously & Poor self-rated health & Having symptoms that impair functioning


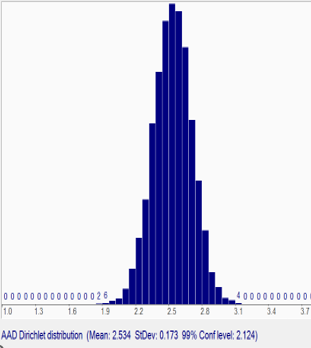


Smoking more than 20 cigarettes & Detrimental factors at work: not dirty & No musculoskeletal diseases & Satisfied with life & Having symptoms that impair functioning


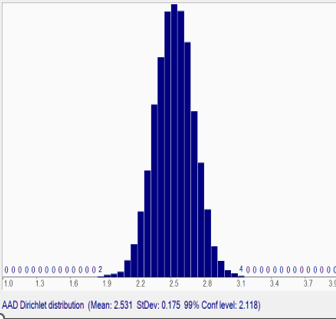


Smoking more than 20 cigarettes & Satisfied with life & Having symptoms that impair functioning & Working hours is 1 or 2 hours & Use of medication


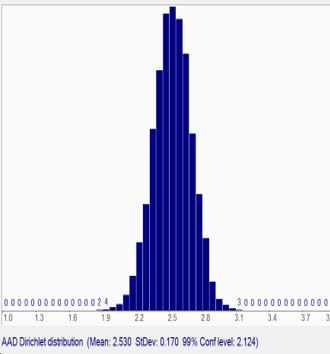


Smoking more than 20 cigarettes & Not changing work due to diseases & Having pain & Smoked previously & Poor self-rated health


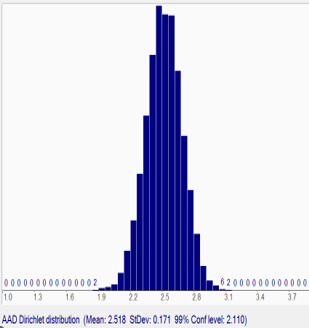


Smoking more than 20 cigarettes & Not changing work due to diseases & Having symptoms that impair functioning & Working hours is 1 or 2 hours & Use of medication


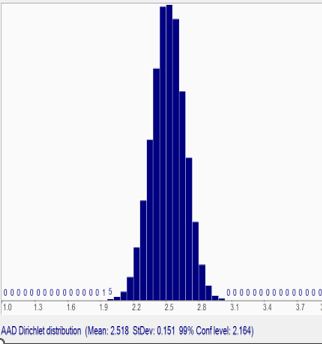


Smoking more than 20 cigarettes & Poor self-rated health & Having symptoms that impair functioning & Working hours is 1 or 2 hours & Use of medication


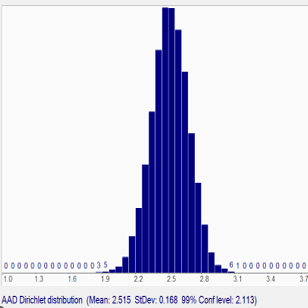


Smoking more than 20 cigarettes & Not changing work due to diseases & Satisfied with life & Having symptoms that impair functioning & Working hours is 1 or 2 hours


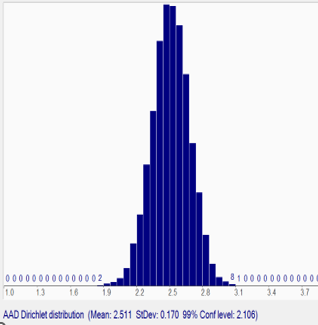


Smoking more than 20 cigarettes & Not changing work due to diseases & Smoked previously & Poor self-rated health & Use of medication


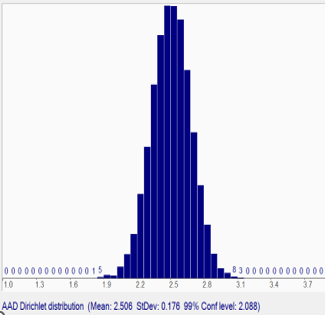


Smoking more than 20 cigarettes & Detrimental factors at work: not dirty & Intention to retire: physical work strain & Having symptoms that impair functioning & Use of medication


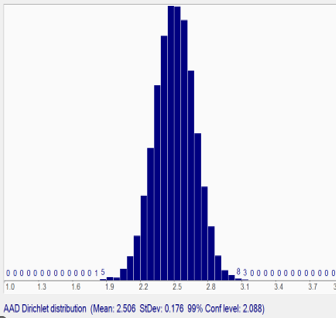


Smoking more than 20 cigarettes & Married & Satisfied with life & Having symptoms that impair functioning & Working hours is 1 or 2 hours
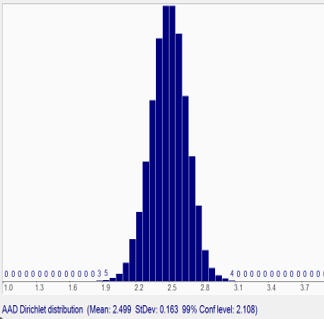


Smoking more than 20 cigarettes & Detrimental factors at work: not dirty & Intention to retire: physical work strain & Satisfied with life & Having symptoms that impair functioning


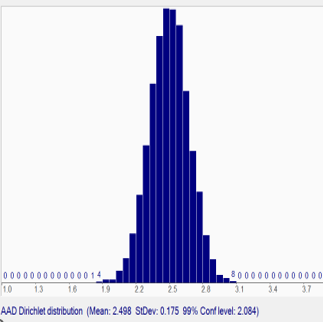


1. Piché, R., Järvenpää, M., Turunen, E. *et al.* Bayesian analysis of GUHA hypotheses. *J Intell Inf Syst* **42**, 47–73 (2014). https://doi-org.libproxy.helsinki.fi/10.1007/s10844-013-0255-6 [↑](#footnote-ref-2)
